# Serum albumin binding knob domains engineered within a V_H_ framework III bispecific antibody format and as chimeric peptides

**DOI:** 10.3389/fimmu.2023.1170357

**Published:** 2023-05-12

**Authors:** Ralph Adams, Callum Joyce, Mikhail Kuravskiy, Katriona Harrison, Zainab Ahdash, Matthew Balmforth, Kelda Chia, Cinzia Marceddu, Matthew Coates, James Snowden, Emmanuel Goursaud, Karelle Ménochet, Jean van den Elsen, Richard J. Payne, Alastair D. G. Lawson, Anthony Scott-Tucker, Alex Macpherson

**Affiliations:** ^1^ Early Solutions, UCB Biopharma UK, Slough, United Kingdom; ^2^ School of Chemistry, The University of Sydney, Sydney, NSW, Australia; ^3^ Australian Research Council Centre of Excellence for Innovations in Peptide and Protein Science, The University of Sydney, Sydney, NSW, Australia; ^4^ Early Solutions, UCB Biopharma SA, Braine L’Alleud, Belgium; ^5^ Department of Life Sciences, University of Bath, Bath, United Kingdom

**Keywords:** knob domain, bispecific, albumin, ultralong CDRH3, peptide

## Abstract

**Background:**

Serum albumin binding is an established mechanism to extend the serum half-life of antibody fragments and peptides. The cysteine rich knob domains, isolated from bovine antibody ultralong CDRH3, are the smallest single chain antibody fragments described to date and versatile tools for protein engineering.

**Methods:**

Here, we used phage display of bovine immune material to derive knob domains against human and rodent serum albumins. These were used to engineer bispecific Fab fragments, by using the framework III loop as a site for knob domain insertion.

**Results:**

By this route, neutralisation of the canonical antigen (TNFα) was retained but extended pharmacokinetics *in-vivo* were achieved through albumin binding. Structural characterisation revealed correct folding of the knob domain and identified broadly common but non-cross-reactive epitopes. Additionally, we show that these albumin binding knob domains can be chemically synthesised to achieve dual IL-17A neutralisation and albumin binding in a single chemical entity.

**Conclusions:**

This study enables antibody and chemical engineering from bovine immune material, via an accessible discovery platform.

## Introduction

A subset of bovine antibodies contain an ultralong CDRH3 (1), where a cysteine rich mini-domain or ‘knob domain’ is presented on an anti-parallel β-strand or ‘stalk’ (2–4), which may constitute the entirety of the paratope (5, 6). We have previously shown that the knob domain can function independently of the antibody scaffold to create an autonomous fragment of just 4-6 kDa (6, 7). In our initial study, five out of six knob domains retained activity when separated from the β-stalk (6). These peptides can be produced recombinantly in eukaryote cells or by solid-phase peptide synthesis (SPPS) (8). Additionally, as recently shown, the proximity of the N- and C- termini of the knob domain enables insertion into protein loops, thereby offering a route to engineer valency into polypeptide chains (9). By virtue of these properties, it appears that knob domains can be employed for biochemical and chemical engineering to create novel constructs.

In this study, we show that, despite an abundance of disulfide bonds, antigen specific knob domains can be isolated by established phage display protocols. Previous discovery methods for ultralong CDRH3 have relied upon cell sorting of antigen specific memory B-cells in tandem with next generation sequencing (6). Yeast or mammalian display with a conserved heavy and light chain pairing have also been successfully employed to discover ultralong CDRH3 against the epidermal growth factor receptor (10) and SARS-CoV receptor binding domains (11). The presence of an endoplasmic reticulum in these eukaryote display systems mirrors the environment in the native expression system and may aid expression.

With the goal of deriving binders that could be employed for *in vivo* half-life extension of low molecular weight pharmaceutical agents, molecules which are typically rapidly excreted via renal filtration, we immunised bovines with human and mouse serum albumin. Binding to serum albumin capitalises on the interaction of albumin with the neonatal Fc receptor (FcRn), which salvages gamma immunoglobulins and serum albumin from lysosomal catabolism thereby conferring a substantial half-life extension to both proteins (12, 13). This approach has been used in a range of FDA approved peptide drugs, including the albumin-binding small molecule paclitaxel (14) and the glucagon-like peptide-1 agonist semaglutide, which contains an albumin-binding fatty acid moiety (15).

From these immunisations, knob domains which bound to human and rodent albumins enabled engineering of chimeric bispecific Fab fragments, via grafting into the heavy chain variable region (V_H_) framework III loop of a TNFα neutralising humanised Fab. These knob domains were also chemically synthesised and refolded with an IL-17A neutralising peptide *in situ*. Herein, we describe the discovery and characterisation of these novel constructs.

This study presents a readily accessible discovery platform for knob domains and highlights the broad utility of these small, antibody-derived binding domains for both recombinant protein and chemical engineering.

## Results

### Discovery of knob domain peptides by phage display

An adult Holstein Friesen cow was immunised with human and mouse serum albumin, which engendered a robust immune response (terminal serum titre of 1/10,000 [data not shown]). Reverse transcriptase PCR was used to prepare cDNA from tissues from the spleen, lymph node and peripheral blood mononuclear cells. One challenge for display is to isolate ultralong CDRH3 sequences, which only constitute around 10% of the repertoire. This has previously been achieved for yeast display by using primers which target a conserved partial duplication of the V-gene segment, which gives rise to a TTVHQ motif in the ascending stalk (10, 16). While this approach is elegant, somatic hypermutation creates diversity in the ascending stalk which may theoretically not be captured by these primers, as evidenced by various ascending stalk motifs in published structures which deviate from TTVHQ (2–4). Therefore, to isolate ultralong CDHR3 DNA for phage display, we developed panels of primers which targeted the ascending and descending stalks based on previous bovine CDRH3 deep sequencing data sets. These primers were designed to target the base of the ascending and descending stalk, adjacent to the conserved frameworks, which should ensure specificity for ultralong CDRH3.

Ultralong CDRH3 sequences amplified in this manner were cloned into a phagemid vector to enable direct display on the PIII protein of M13 Phage. After electroporation of competent bacteria, the library was estimated to contain 1x10^9^ displaying phage, based on limiting dilution experiments. Three rounds of selection against human or mouse serum albumin converged on single human serum albumin binder, aHSA (CDRH3 sequence [knob domain underlined]: TTVHQQTHQDQTCPDGYTRTNYYCRRDGCGSWCNGAERQQPCIRGPCCCDLTYRTAYEYHV and enriched for mouse serum albumin binder, aMSA (CDRH3 sequence [knob domain underlined]: TTVHQRTKTTCPDGQRDRGGCSGPYSCGGDNCCAYAAASVYRGYSCKDTYEWYVDT. Alternating selections against mouse and human serum albumins did not yield cross reactive binders in this study.

**Figure 1:**
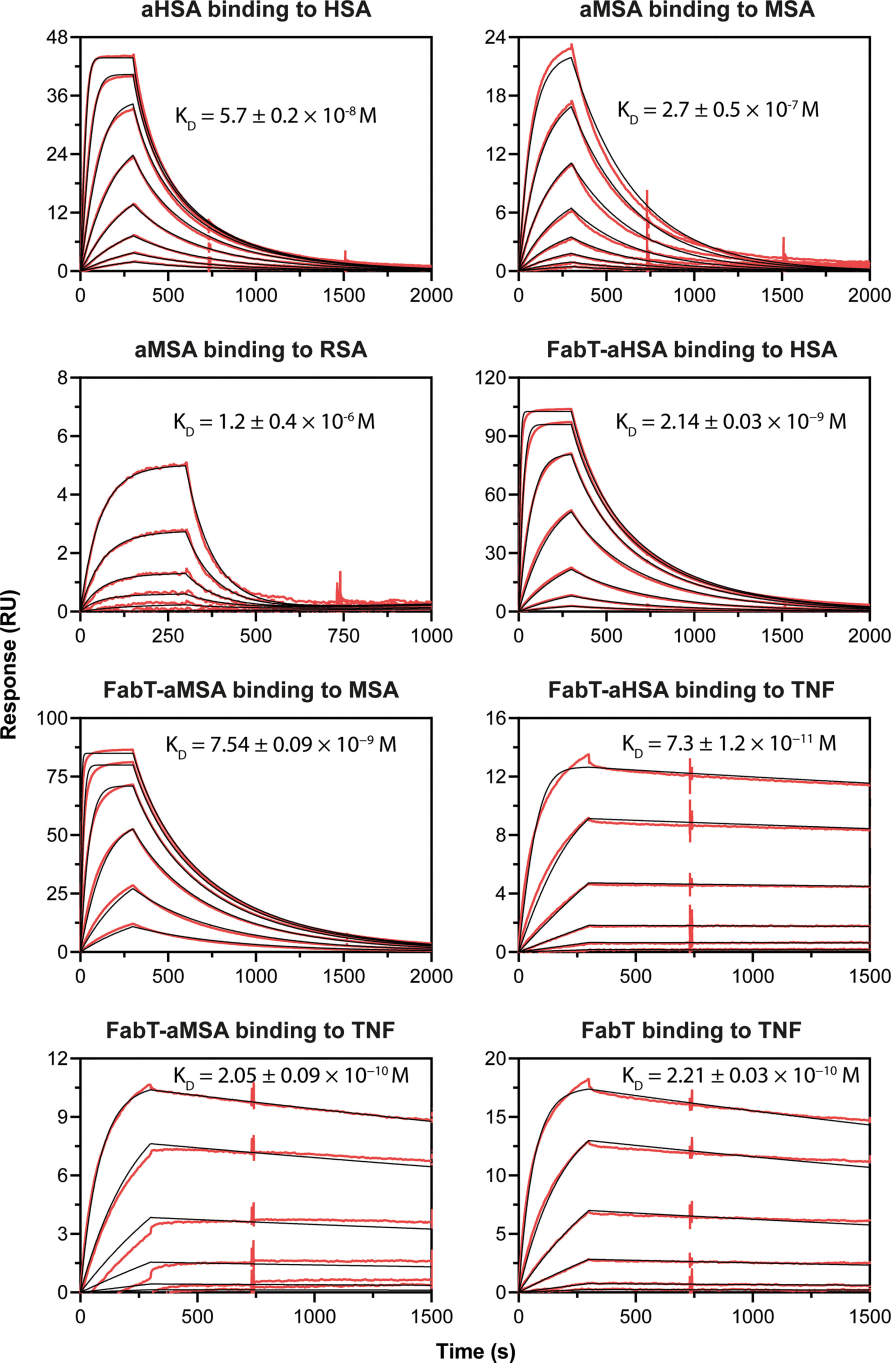
Biacore SPR data for the aHSA and aMSA peptides and FabT framework III fusions. Biacore SPR data is shown for the aHSA and aMSA peptides. Equilibrium dissociation constants (K_D_) are derived from a 1:1 binding model and are reported as a mean of *n=3* multi-cycle kinetics experiments with error shown as standard deviation.

The knob domain sequences of aHSA and aMSA were prepared by SPPS and antigen binding was characterised by multi-cycle kinetics surface plasmon resonance (SPR) experiments, using a Biacore 8K (**Figure 1**; **Table 1**). This revealed aHSA bound human serum albumin (HSA) with an equilibrium dissociation coefficient (K_D_) of 57 nM, with no binding to rat or mouse serum albumins detected. Conversely, aMSA bound mouse and rat serum albumin, with a K_D_ of 270 nM (mouse) and 900 nM (rat) but did not display binding to HSA. It has previously been shown that, due to the abundance of serum albumin, that even µM affinity ligands are able to achieve half-life (t_1/2_) extensions equivalent to the t_1/2_ of serum albumin for small antibody fragments *in vivo (* 17). We therefore hypothesised that these knob domains could effectively extend t_1/2_ when suitably engineered into constructs which exhibit short exposure.

**Table 1:** Antigen-binding kinetics of the aHSA and aMSA knob domains. Errors are standard deviations (n ≥ 3).

Knob domain	Albumin species	*k*_a_, M^-1^s^-1^	*k*_d_, s^-1^	*K*_D_, M
aHSA	Human	1.1 ± 0.5 × 10^5^	6 ± 3 × 10^-3^	5.7 ± 0.2 × 10^-8^
	Cyno	No binding		
	Mouse	No binding		
	Rat	No binding		
aMSA	Human	No binding		
	Monkey	No binding		
	Mouse	1.06 ± 0.12 × 10^4^	2.83 ± 0.17 × 10^-3^	2.7 ± 0.5 × 10^-7^
	Rat	1.1 ± 0.3 × 10^4^	1.21 ± 0.12 × 10^-2^	1.2 ± 0.4 × 10^-6^

### Engineering the V_H_ framework III loop of a humanised Fab fragment

We have previously reported that knob domains can be readily incorporated into protein loops to introduce valency to polypeptide chains (9) and noted the potential suitability of the mammalian V_H_ framework III, or non-variable CDR4, loop as an insertion site, whereby binding to the canonical Fab antigen might be retained but a knob domain could be introduced to produce a proximally constrained bi-specific molecule, with the potential for additional stabilisation of the knob domain termini by the V_H_ framework. We selected a humanised Fab fragment, FabT, which binds and potently neutralises human TNFα as an acceptor scaffold. The two knob domains were inserted between residues A75 and K76 of VH framework III. Single glycine residues were included at both the N- and C-termini of each insertion to act as spacer residues. These constructs were expressed in high yield following transient transfection in CHO S-E cells (>0.1 g/L), with no evidence of significant quantities of covalent aggregate.

In SPR multi-cycle kinetics experiments (**Figure 1**; **Table 2**), FabT-aHSA bound HSA with a K_D_ of 2.1 nM and TNFα with a K_D_ of 73 pM. FabT-aMSA bound mouse serum albumin (MSA) with a K_D_ of 7.4 nM and TNFα with a K_D_ of 205 pM. The unmodified FabT fragment bound TNF with a K_D_ of 221 pM and did not bind either HSA or MSA (data not shown). Insertion of these two knob domains into framework III appears to increase their affinity for serum albumin, relative to the isolated domains, and may indicate that the V_H_ framework offers a stabilising function, which is analogous to the native β-stalk.

**Table 2:** Antigen-binding kinetics of FabT-aHSA, FabT-aMSA and FabT. Errors are standard deviations (*n =3*).

Fab	Antigen	*k*_a_, M^-1^s^-1^	*k*_d_, s^-1^	*K*_D_, M
FabT-aHSA	HSA	1.82 ± 0.02 × 10^6^	3.89 ± 0.02 × 10^−3^	2.14 ± 0.03 × 10^−9^
	MSA	No binding		
	TNF	6.4 ± 0.8 × 10^5^	4.7 ± 1.4 × 10^−5^	7.3 ± 1.2 × 10^−11^
FabT-aMSA	HSA	No binding		
	MSA	4.09 ± 0.13 × 10^5^	3.08 ± 0.06 × 10^−3^	7.54 ± 0.09 × 10^−9^
	TNF	4.06 ± 0.06 × 10^5^	8.3 ± 0.5 × 10^−5^	2.05 ± 0.09 × 10^−10^
FabT	HSA	No binding		
	MSA	No binding		
	TNF	4.47 ± 0.15 × 10^5^	9.9 ± 0.3 × 10^−5^	2.21 ± 0.03 × 10^−10^

**Figure 2:**
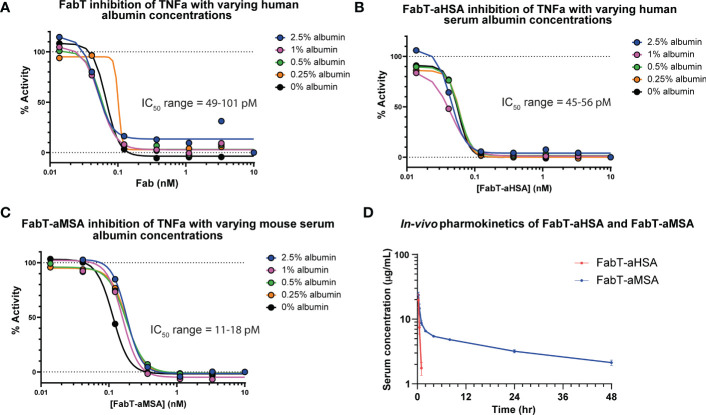
*In vitro* cellular pharmacology and *in vivo* pharmacokinetics of FabT constructs. Inhibition of TNFα in a Nf-κB reporter gene assay is shown for FabT, FabT-aHSA and FabT-aMSA in panels **(A–C)**, respectively. Varying the concentrations of serum albumin did not substantially affect the IC_50_ values, suggesting that there is minimal competition for the antibody paratope between the two antigens. **(D)** shows the FabT-aHSA and FabT-aMSA serum concentrations following intravenous injection at 2 mg/kg to Balb/c mice (*n=3*, dots are mean ± standard deviation). Of note, FabT-aMSA shows a marked extension in serum half-life, relative to FabT-aHSA, mediated by binding to mouse serum albumin.

The Fab constructs were tested in a reporter gene assay which measured activation of Nf-κB in response to TNFα stimulation (**Figure 2**). Both FabT-aHSA and FabT-aMSA were equipotent to the parent FabT which, viewed in the context of comparable binding data, indicates that insertion of the knob domain into framework III was not detrimental to binding of the canonical antigen. To assess if there was competition upon concomitant binding of TNFα and serum albumin, experiments were performed where the albumin concentration of the assay was varied from 0 – 2.5% (*w/v*). The albumin concentration did not affect the potency of TNFα inhibition even at 2.5% (*w/v*) serum albumin (*ca* 379 µM), which is in excess of one thousand-fold above K_D_.

Next, we sought to measure the *in vivo* pharmacokinetics (PK) of the two Fab fragments to ascertain if a t_1/2_ extension had been achieved (**Figure 2**; **Table 3**). The FabT-aHSA and FabT-aMSA Fabs were administered intravenously to BALB/c mice and, following lysis of whole serum, drug serum concentrations were quantified by liquid chromatography mass spectrometry (LC-MS), using a signature peptide that was unique to the human Fab fragments, and subjected to a two-compartment analysis. Following intravenous injection, FabT-aHSA only remains in the systemic circulation for a short period of time, as expected due to the molecular weight of the Fab. The central volume of distribution was 34 mL/kg. Clearance from the central compartment was close to the glomerular filtration rate in mice at 136 mL/hr/kg. Half-life was short at 0.2 hr. In comparison, serum concentrations of FabT-aMSA followed a biphasic decay over time. Steady state volume of distribution was 285 mL/kg. Elimination clearance was moderate at 7.9 mL/hr/kg, resulting in a half-life of 27 hr, in line with the half-life of mouse albumin in mouse (18). Ultimately, these data indicated that half-life extension had been achieved.

**Table 3:** Pharmacokinetic parameters measured for FabT-aHSA and FabT-aMSA following intravenous injection to Balb/c mice at 2 mg/kg (n=3; Values are mean ± SD).

Parameter	Unit	FabT-aHSA	FabT-aMSA
CL	mL/hr/kg	136 ± 35	7.9 ± 0.8
V_ss_	mL/kg	34 ± 9	285 ± 3
T_1/2_	hr	0.2 ± 0.003	27 ± 3
MRT_all_	hr	0.2 ± 0.006	17.4 ± 0.2
AUC_all_	μg.hr/mL	145 ± 3	187 ± 9

### Structural characterization

**Figure 3:**
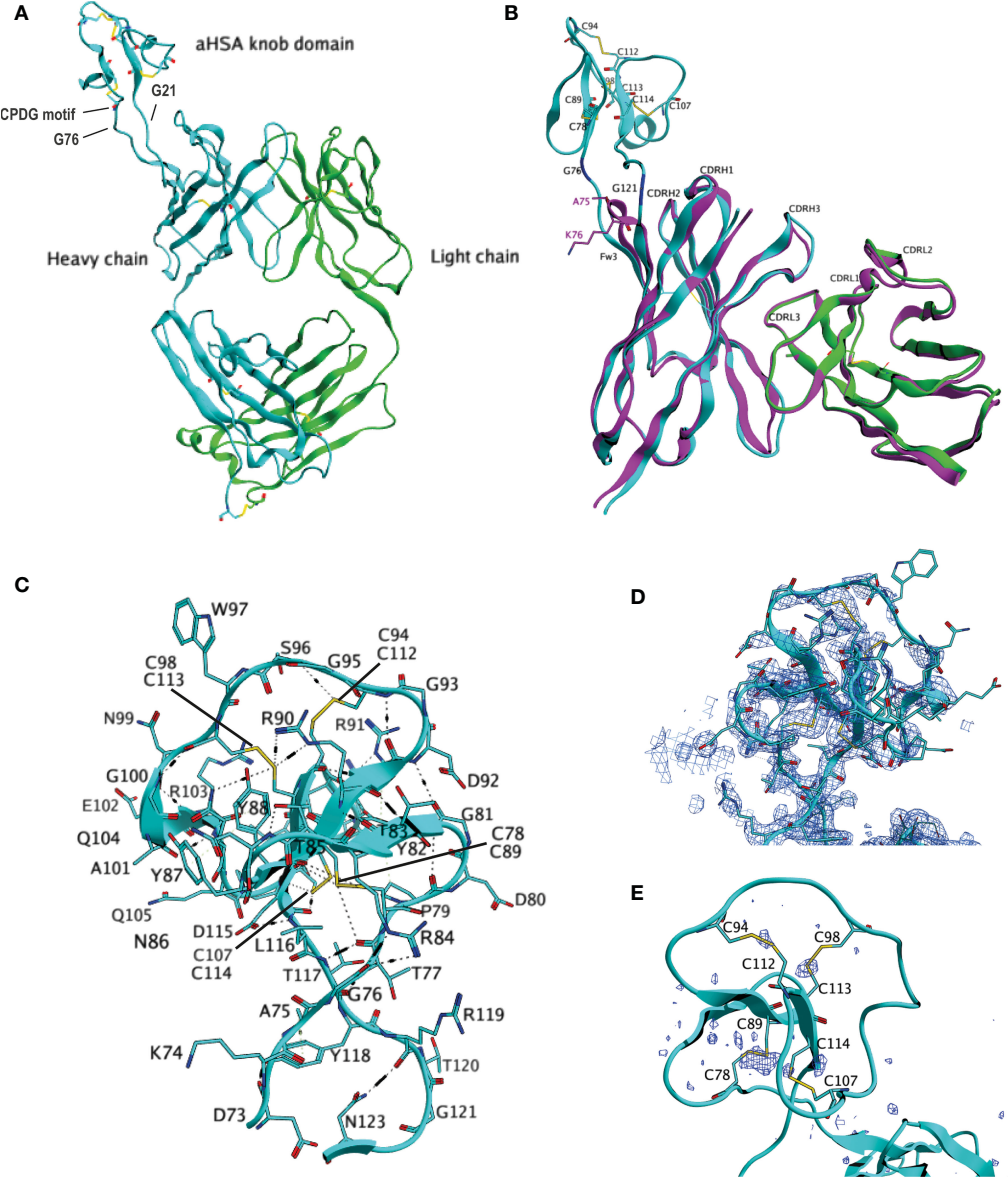
Crystal structure of FabT-aHSA. **(A)** shows the crystal structure of FabT-aHSA. The heavy chain is shown in cyan, light chain in green. **(B)** shows the superposition of FabT-aHSA CDRs onto those of FabT, with the FabT-aHSA V_H_ shown in cyan, V_L_ in green and FabT V_H_V_L_ in magenta. The electrostatic and disulfide bond interactions of the aHSA knob domain are shown in **(C, D)** shows a 2Fo-Fc electron-density map contoured at 1.0σ above mean for the aHSA knob domain. Correct building of the disulfide bond network was confirmed using sulfur-SAD, peaks of the anomalous difference map, contoured at 3.0σ above mean, are shown in dark blue in **(E)**.

As both FabT-aHSA and FabT-aMSA retained affinity for TNF, we hypothesized that the insertion of the respective knob domains into framework III had little or no impact on the structure of the Fab. To determine whether the CDR conformations of FabT-aHSA and FabT-aMSA align with those of FabT, attempts were made to crystallize all three proteins. Conditions for crystallization were identified for FabT and FabT-aHSA but not for FabT-aMSA. FabT crystallized in the space group P1 2_1_ 1 with three copies in the asymmetric unit. The structure was determined by molecular replacement using the coordinates of an unpublished in-house Fab (**Figure 3**). It was refined to 1.7 Å with R_work_/R_free_ = 0.1662/0.1943 (see **Table S1**) and deposited in the PDB (ID: 8C7V). FabT-aHSA crystallized in the space group P2_1_ 2_1_ 2_1_ with a single copy in the asymmetric unit. The structure was determined by molecular replacement using the coordinates of the refined structure of FabT. It was refined to 2.0 Å with R_work_/R_free_ = 0.1859/0.2257 (see **Table S1**) and deposited in the PDB (ID: 8C7J). Superposition of the CDRs of FabT-aHSA onto those of FabT resulted in Cα root mean squared deviation (RMSD) of 0.54 Å. These structural data confirmed that insertion of the knob domain into Framework III had little or no impact on the structure of the Fab.

The knob domains had been inserted between residues A75 and K76. This is the tip of the loop D73-N77 that connects two β-sheets. The structure of FabT-aHSA showed that, following insertion of the knob domain into Framework III, the spatial positions of D73 and N123 (equivalent to N77 in the parent Fab) remain unaffected. Our results show that this is key to preserving the immunoglobulin fold since the hydrogen bonds to S124 (equivalent to S78) and A24, respectively, in the flanking β-sheets are intact. In contrast, K74, A75 and K76 underwent large movements of 4.8 Å, 4.4 Å and 6 Å for the Cα atoms, respectively. K74-T77 and L116-K122 (the latter equivalent to K76) form a short, twisted stalk which is stabilized by multiple hydrogen bonds. There is a hydrogen bond network formed by T77 with T117, R84 and R119. There are 2 further hydrogen bonds between N123-R119 and D73-Y118. C78-D115 forms a large globular domain containing 3 anti-parallel β-sheets (Y82-T85, Y88-R91 and C113-D115) and a short α-helix (G100-R103) which are connected by 4 loops. Disulfide bond C78-C89 anchors the globular domain to the stalk. At the core of the globular domain, there are 3 three consecutive cysteine residues, C112, C113 and C114, that form disulfide bonds with C94, C98 and C107, respectively. These 3 disulfide bonds radiate from the centre to constrain the outer loops. Akin to other knob domain or ultralong CDRH3 Fab structures (2–5), the disulfide bonding pattern within aHSA is not consistent with the conventional classifications of cystine-knot (growth factor cystine-knots, inhibitor cystine-knots or cyclic cystine-knots), as found in certain small cysteine-rich toxins and growth factors (19). Extensive hydrogen bonding further stabilizes the knob domain, which could aid correct formation of the disulfide bonds during folding.

To build and refine a Fab structure following molecular replacement with the coordinates of another Fab is relatively straightforward given the conservation in structure between Fabs. In contrast, knob domains are highly heterogeneous and consequently there are no suitable coordinates for molecular replacement. For FabT-aHSA, the electron density of the knob domain was comparatively weaker than that of the Fab domain (see **Supplementary Figure S3**). This may reflect movement of the knob domain relative to the Fab due to the linker glycine residues. Furthermore, loops not constrained by internal contacts or crystal contacts with molecules in neighboring asymmetric units, may be mobile and show weaker density.

The knob domain occupied a solvent channel and exhibited high local B-factors, relative to the rest of the structure. To confirm the accuracy of the manual modelling of the knob domain disulfide bonds, an orthogonal sulfur-SAD (single-wavelength anomalous diffraction) dataset was collected on a second crystal of FabT-aHSA. This technique can detect the anomalous signal from sulfur and locate sulfur containing residues cysteine and methionine. Chloride atoms also emit an anomalous signal at this wavelength and can be detected in the derived anomalous difference map.

**Supplementary Figure S2** shows the FabT-aHSA structure overlayed with the anomalous difference map. Strong signals overlay the interchain disulfide bonds in V_H_ (C), V_L_ (D), CH1 (E), CK (F), all 3 methionine residues present in the Fab and likely chloride atoms present in the mother liquor. In contrast, the signal corresponding to the interchain disulfide bond is low. The electron density for these residues is weaker than for the rest of the Fab indicating mobility in this region. This is a common feature of Fab structures where this disulfide bond can often not be resolved. In the knob domain (**Figure 3**), there is a strong signal overlaying disulfide bond C78-C89. Good signals overlay the other 3 three modelled disulfide bonds, C94-C112, C98-C113 and C107-C114. The strength of these 3 signals all exceeds that of the Fab interchain disulfide bond. The alignment of the signals in the anomalous difference map with all four disulfide bonds support the modelled knob domain structure.

**Figure 4:**
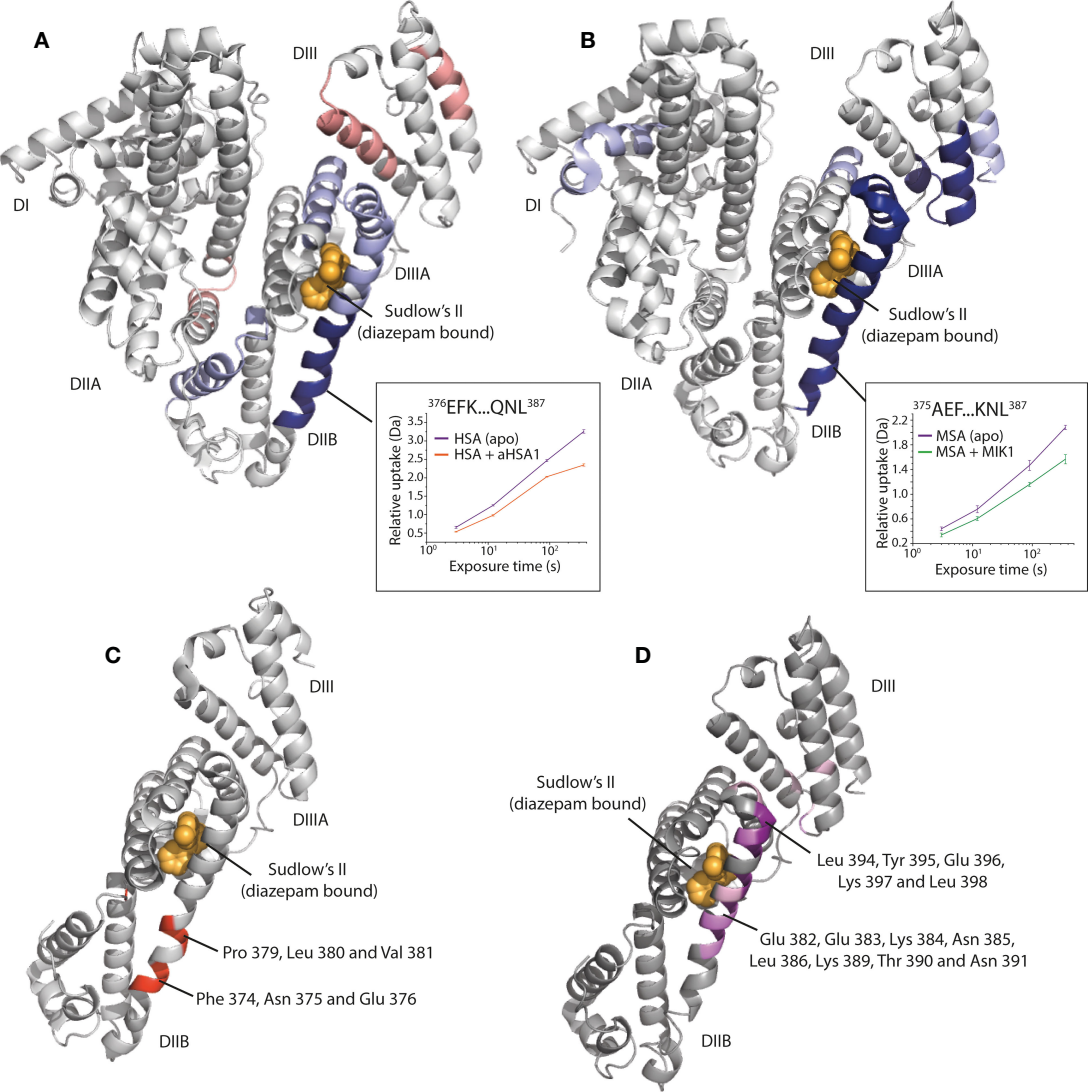
Epitope mapping of the aHSA and aMSA binding sites on serum albumin. **(A)** shows HDX data for aHSA coloured onto a human serum albumin crystal structure (PDB: 1AO6) (20). Sudlow’s site II with diazepam bound (PDB: 2BXF) (21) is shown for reference, diazepam was not used in the experiments and is shown to illustrate the position of Sudlow’s II. Areas of solvent protection and deprotection are coloured blue or red, respectively, by relative intensity. A clear area of protection suggests the epitope is present within the DIIB domain, adjacent to Sudlow’s II. **(B)** shows HDX data for aMSA coloured onto an AlphaFold (22) model of MSA, where a clear area of protection is again visible on the DIIB domain. **(C, D)** show the position of scanning alanine mutations on the DII/DIII domains of HSA and MSA, respectively. Mutations which attenuated binding of the FabT knob domain fusion proteins are in shown in red on HSA for FabT-aHSA and in purple on MSA for FabT-aMSA. These areas are contained within the areas of solvent protection as predicted by HDX.

Hydrogen deuterium exchange mass spectrometry (HDX-MS) was used to derive epitope information for both aHSA and aMSA. These experiments revealed narrow areas of protection as putative, and potentially overlapping, epitopes on domain IIB of HSA and MSA for aHSA and aMSA, respectively (**Figure 4**). In accordance with our PK data for FabT-aMSA, these epitopes are distal to the neonatal Fc receptor (FcRn) binding site (23) and should therefore not sterically compete with FcRn. The epitopes are also more than 25Å from the multi-metal binding site on domain IIA. However, based on the available structures (24), knob domain binding may fully or partially obscure drug binding site II (Sudlow’s site II) which is responsible for binding Ibuprofen, halothane, and diazepam and, in combination with drug binding site I (Sudlow’s site I), thyroxine, and the uremic toxins indoxyl sulfate and 3-carboxy-4-methyl-5-propyl-2-furanpropanoic acid (CMPF) (21, 25). However, given the very small percentage of albumin which would be involved in half-life extension of a potential therapeutic, any impingement of carrier function is likely to be low.

To validate the epitope information from our HDX experiments, domain mapping experiments were performed, whereby individual or pairs of domains from HSA and MSA were expressed and purified. Binding to each domain was then assessed in Octet bilayer interferometry experiments. While the DII domain could not be expressed in isolation, these experiments confirmed binding against the DII/DIII portion of albumin (Data not shown). To obtain amino acid resolution of the epitope, panels of alanine HSA and MSA mutants were designed, across the DII and DIII domains, at positions which were predicted to maintain the helical propensity of the region. Bilayer interferometry was used to measure binding affinity for each mutant to assess the contribution of individual residues to the epitope. For both FabT-aHSA and FabT-aMSA, the attenuating mutations were contained within the solvent protected regions identified in our HDX experiments (**Figure 4**).

For FabT-aHSA, likely interacting residues comprise Phe 374, Asp 375, Glu 376, Pro 379, Leu 380 and Val 381 of HSA. The area of solvent protection surrounding Sudlow’s site II, which was observed in response to aHSA binding in our HDX experiments, was not affected by single alanine mutations. This may indicate that a conformational change is induced in this nearby region upon binding of aHSA.

For FabT-aMSA, attenuating mutations span a larger area on MSA which surrounds Sudlow’s site II, comprising Glu 382, Glu 383, Lys 384, Asn 385, Leu 386, Lys 389, Thr 390, Asn 391, Leu 394, Tyr 395, Glu 396, Lys 397 and Leu 398. Additionally, two moderately attenuating mutations were found on DIII, which may suggest further epitope interactions outside of DIIB.

A comparative sequence analysis of the attenuating mutations reveals differences which may explain the lack of species cross reactivity, notably a lack of conservation of Phe 374 and Asp 375 in rodent albumins and Lys 378 in albumin from cynomolgus monkeys. The putative MSA epitope also comprises several changes (Lys 395, Val 388, Thr 390, Asp 393, Tyr 395 and Lys 397) which likely explain the selectivity of aMSA for rodent albumins.

### Creation of chimeric peptides

Having recombinantly engineered a Fab fragment we next chemically engineered chimeric peptides. A published ‘HAP’ peptide, which has a simple linear sequence (IHVTIPADLWDWINK) was used as an inhibitory payload for the aMSA and aHSA knob domains. The HAP peptide binds to the IL-17A homodimer with nM affinity, to sterically prevent association with IL-17 Receptor A (IL-17RA) and ablate signalling (26).

The HAP peptide was appended to the N-terminal of the knob domain on a diethylene glycolate (PEG2) linker during SPPS. To ensure correct disulfide bond formation, the knob domain was refolded with the HAP peptide *in situ*. Following a preparative reversed phase chromatography step the resulting peptides were subjected to LC-MS analysis (S8). The chimeric peptides aHSA-HAP and aMSA-HAP were obtained with a 1.1% and 2.4% yield, respectively, with a 98% average yield per step. While final yields were acceptable, we note that they are slightly below recently reported yields for V_HH_ chemical syntheses (27, 28).

**Figure 5:**
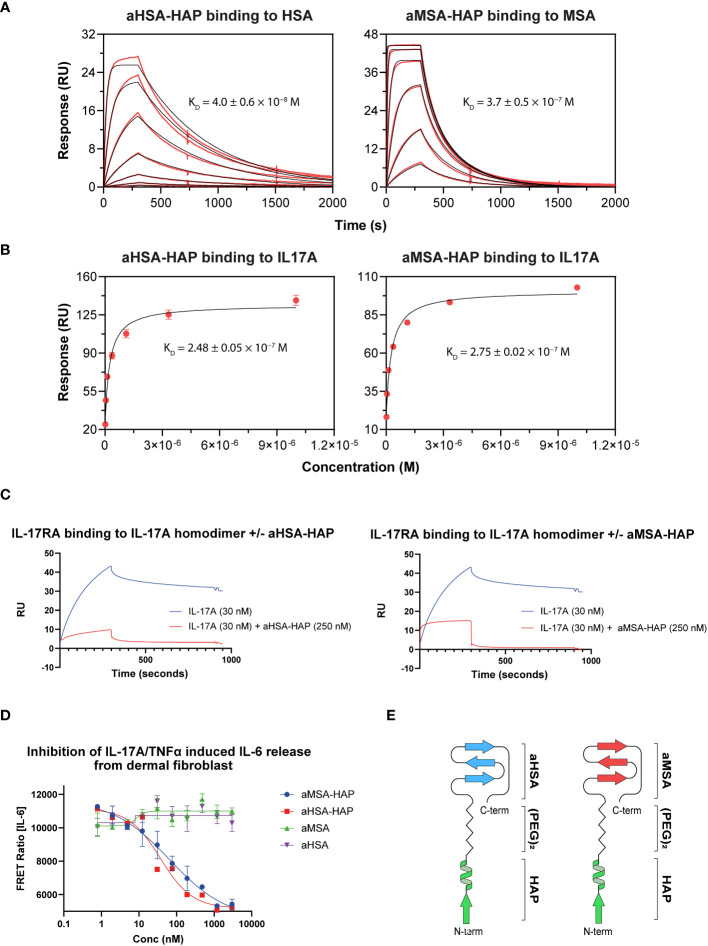
Creation of chimeric peptides. **(A)** shows binding of aHSA-HAP and aMSA-HAP chimeric peptides to HSA and MSA, respectively, by Biacore. Binding to IL-17A is shown in **(B)** with data fitted using a steady state model. Binding affinities are displayed as mean of *n=3* experiments with errors shown as standard deviation. **(C)** shows a competition assay whereby the presence of 250 nM of chimeric peptide ablates binding of IL-17A to IL-17RA. Dose dependent inhibition of IL-6 release from dermal fibroblasts, in response to dual IL-17 and TNFα stimulation, is shown in **(D)**. A schematic showing the structures of the chimeric peptides are shown in **(E)**.

In SPR multi-cycle kinetics experiments, aHSA-HAP bound HSA with a K_D_ of 40 nM while aMSA-HAP bound MSA with a K_D_ of 370 nM, equivalent to the isolated albumin binding peptides (**Figure 5**; **Table 4**). We observed complex kinetics for the HAP peptide chimeras when binding to IL-7A, which were not well described with a 1:1 binding model, and we therefore opted to derive equilibrium dissociation constants using steady state fitting. Under steady state conditions, the aHSA-HAP and aMSA-HAP bound IL-17A with K_D_ of 248 nM and 275 nM, respectively (**Figure 5**; **Table 4**). There was no evidence of aHSA or aMSA binding to IL-17A (data not shown).

**Table 4:** Binding of aHSA-HAP and aMSA-HAP.

	Ligand	*k*_a_, M^-1^s^-1^	*k*_d_, s^-1^	*K*_D_, M
aHSA-HAP	HSA	2.0 ± 0.4 × 10^5^	8.1 ± 0.5 × 10^−3^	4.0 ± 0.6 × 10^−8^
	MSA	No binding		
	IL-17A	n.d.	n.d.	2.48 ± 0.05 × 10^−7^
aMSA-HAP	HSA	No binding		
	MSA	4.5 ± 0.7 × 10^3^	1.66 ± 0.05 × 10^−3^	3.7 ± 0.5 × 10^−7^
	IL-17A	n.d.	n.d.	2.75 ± 0.02 × 10^−7^

The binding affinities to IL-17A were determined by steady-state analysis therefore rate constants are not reported. Errors are standard deviations (n ≥ 3).

The chimeric HAP peptides were also tested in an SPR competition assay, where IL-17A was passed over immobilised IL-17RA. In the presence of a single concentration of HAP peptides (250 nM) the interaction was substantially abrogated (**Figure 5**). Finally, to measure potency, serial dilutions of the chimeric HAP peptides were tested in a fibroblast IL-6 release assay, where IL-17A potently synergises with TNFα to drive IL-6 release. The aHSA-HAP inhibited IL-6 release with an IC_50_ of 81 nM and the aMSA-HAP inhibited with an IC_50_ of 38 nM (**Figure 5**). These data show that IL-17A binding and inhibition is present in a single, albumin binding chemical entity.

## Discussion

This study highlights the utility of knob domains as tools for chemical and recombinant protein engineering. In our antibody engineering experiments, we show that the V_H_ framework III loop can be a viable site for grafting knob domain peptides to create bispecific or potentially biparatopic antibodies.

We have used phage display to enrich for antigen binding knob domains. Surprisingly, given the disulfide rich nature of the domains, we show that phage display is a viable route to discover knob domains. Display of cysteine rich peptides is non-trivial with a recent study estimating that approximately 17% of a cysteine rich peptide library was displayed in a mammalian display system (29). The ability of knob domains to be isolated by phage display lowers the barrier of entry for working with bovine ultralong CDRH3, given the established nature of the technique. We additionally present a panel of primers that have been designed to allow selective amplification of ultralong CDRH3 sequences (S8), which may be used in a range of different discovery applications.

We note that, despite the robust immune response, the knob domains presented in this study were of comparably modest affinity. This may reflect either a bias in the immune response, whereby shorter conventional CDRH3 were favoured for this particular antigen, or a bias in the system, whereby phage display may not always converge on the highest affinity ultralong CDRH3 sequence, but instead favours sequences which are best expressed on the pIII protein. Alternatively, we cannot preclude that high affinity knob domains were not amplified by the stalk specific primers, particular given the low coverage (~10%) that we observed testing the primer sets against the cell sorted B-cells from the albumin immunisation, which was markedly lower than for the other antigen enriched training sets.

Also of note was that the knob domain used in this study displayed increased affinity when formatted as framework III insertions, this may arise due to the V_H_ conferring additional stability to the N- and C- termini, in a manner analogous to the native bovine β-stalk, which has been shown to confer increased thermal stability to bovine antibodies and modest increases in binding affinity (30). Similar increases in affinity have also been observed in certain head-to-tail cyclised knob domains (8), which may indicate a potential benefit of constraining the knob domain termini during reformatting.

By virtue of their small size, knob domains are amenable to chemical synthesis, and we have shown synthesis of chemical knob domain chimeric peptide, this approach permits bovine immune system diversity to be utilised for peptide design and enables bispecific peptide constructs to be engineered. These albumin binding constructs, while larger than conventional peptide drugs, avoid fatty acid conjugation which may introduce additional hydrophobicity.

## Methods

### Bovine immunization

One adult Friesian cow was immunized with a 1:1 mixture of full-length purified human serum albumin (HSA, Stratech 169598) and mouse serum albumin (MSA, Stratech A1274-90J). 1.0 mg total protein of protein at a concentration of 2 mg/mL was emulsified 1:1 (v/v) with Montanide ISA 71 VG Adjuvant (SEPPIC) in a total volume of 1 mL. Three subcutaneous injections into the shoulder were performed at 1-month intervals. Ten days after each immunization, 10 mL of blood was taken to allow testing of the serum antibody titre.

### Harvesting of immune material

Five-hundred-millilitre samples of whole blood were taken, post immunisation with HSA and MSA. PBMCs were isolated using Leucosep tubes (Griener Bio-one), as per the manufacturer’s instructions. After immunisation, the animals were euthanized, and a draining lymph node from the neck—adjacent to the site of immunisation—and a portion of spleen were collected. The tissues were homogenised using a gentle MACS tissue dissociator (Miltenyi), passed through a 40 μm cell strainer, and collected in Roswell Park Memorial Institute (RPMI) 10% foetal calf serum (FCS). Cells were frozen in FCS, 10% dimethyl sulfoxide.

### Primer design

Working with five next generation sequencing datasets, sequence data were processed to enable design of stalk specific primer panels. Briefly, five bovine immunisations were conducted with human complement C5 (immunisation protocol as described in (6)), green fluorescent protein, human IL-2 and human and mouse serum albumins (immunisation protocol as described herein). Cell sorting of antigen positive B-cells from lymph nodes were performed as previously described (6). Following RT PCR, primers against the conserved V_H_ framework III and IV regions were used to amplify CDRH3 sequences, irrespective of length (6). The primers used were 5′-GGACTCGGCCACMTAYTACTG-3′ and 5′-GCTCGAGACGGTGAYCAG-3′, while the RT PCR and PCR protocols were as described in S7. The next generation sequencing of the CDRH3 sequences from the PCR products was performed at Genewiz (Azenta Life Sciences) using their AMPLICON-EZ service, except for human complement C5, which was subjected to ion torrent sequencing as described in (6). Paired end reads which shared an overlapping sequence to enable pairing to form a combined sequence read of ~300 base pairs (bp) were taken forward as putative sequences, with ultralong CDRH3 constituting 5-10% of the returned sequences.

The ascending stalk region was identified as the start of the CDR3 (Kabat position 93) to the first cysteine. The descending stalk was identified as the region from the last cysteine in CDRH3 to the next aromatic residue (Phe/Tyr/Trp/His). Each ascending stalk sequence was then compared against the set of 10 primers using an in-silico binding prediction process that scored mismatches based on their proximity to the 3’ of the sequence, based on the known importance of 3’ binding compared to binding at 5’ end of the sequence. To quantify this, a mismatch between the primer and sequence was given a score using its position number in the sequence and the cube of that value. For example, a single mismatch at position 14 would be scored as 14^3^, which would give this primer a mismatch score of 2744. We hypothesised that if there was a mismatch in the first three positions of the primer (the three base pairs at the 3’ of the sequence) then the primer would not bind. Accordingly, we calculated a cut off using the mismatch score from the position at the 3’ of the sequence. For a 21 base pair primer, a single mismatch at the 3’ end would be calculated as 21^3^, resulting in a cut off score for this primer of < 9261. The cut-off was a cumulative mismatch score across the entire primer sequence and a primer could therefore be deemed to be non-binding due to a single change at the 3’ end or multiple, less significant mismatches across the sequence.

If none of the 10 primers produced a score that was below the cut-off, then the sequence was not considered to be bound by the primer set. A final primer set was designed (refer to S7) which achieved varying coverage across the difference samples from 10-86%. Of note, the lower coverage scores were against the albumin immunisations, were there was significant enrichment of β-stalks which we predicted would not be captured by of our primer sets.

### Phage display

Phage display was performed broadly as previously described (31). Detailed phage display protocols are displayed in the **Supplementary Materials (S7)**.

### Expression and purification of FabT aHSA/aMSA Fabs

DNA encoding the light and heavy chain variable regions of Fab-T, FabT-aHSA and FabT-aMSA were cloned into UCB expression vectors containing DNA encoding human light chain Cκ and human heavy chain γ1 C_H_1 regions, respectively. The Fabs were transiently expressed in CHO-S XE cells (32), a CHO-K1 derived cell line, using electroporation and then purified from culture supernatants by affinity chromatography using a HiTrap Protein G column (GE Healthcare, Buckinghamshire, UK). Following a washing step with PBS (pH 7.4), the bound material was eluted with 0.1 M glycine (pH 3.2) and neutralised with 2 M Tris-HCl (pH 8.5). Fractions containing Fab were pooled, quantified by absorbance at 280 nm, and concentrated using Amicon Ultra-15 10 kDa molecular weight cut off centrifugal filter units (Merck Millipore, Massachusetts, 298 USA). To isolate the monomeric fractions of Fab, we used size-exclusion chromatography over a HiLoad 16/60, Superdex 200 column (GE Healthcare) equilibrated with PBS (pH 7.4). Fractions containing monomeric Fab were pooled, quantified, concentrated to 10 mg/mL for crystallography, and stored at 4°C.

### Biacore SPR

*Antibody binding assays:* The SPR multicycle kinetics measurements were carried out on a Biacore 8K+ instrument at 25°C. To determine the albumin binding kinetics of knob domains and knob domain-FabT fusions, a Biacore CM5 chip was coated with serum albumins from various species via amine coupling to a maximum of 100 response units (0.5 µg/mL in 10 mM sodium acetate, pH 4.0). The chip was then subjected to repeated binding cycles of knob domain serial dilutions in HBS-EP+ buffer (10 mM HEPES pH 7.4, 150 mM NaCl, 3 mM EDTA, 0.005% v/v Surfactant P20). For each injection, a flow rate of 40 µL/min was used. Contact and dissociation times were 300 s and 3600 s, respectively; the surface was regenerated by dissociation in buffer. To determine the TNF binding kinetics of knob domain-FabT fusions, the surface was coated with anti-human Fab antibodies to a maximum of 100 response units (0.5 µg/mL in 10 mM sodium acetate, pH 4.5) and used for capturing FabT. Serial dilutions of TNFα were prepared in HBS-EP+ buffer and injected at 40 µL/min. Association and dissociation were recorded for 300 s and 1200 s, respectively; regeneration was conducted by consecutive washes with 50 mM HCl, 5 mM NaOH and 50 mM HCl, 60 s each. Kinetic rates were obtained by fitting the reference-subtracted data to a 1:1 binding model using Biacore Insight Evaluation Software.

*HAP Peptide binding assay*: Solutions of HSA, MSA and IL-17A were prepared at 2 µg/mL in 10 mM sodium acetate (pH 4.5) were immobilized on a CM5 sensor chip, via amine coupling, resulting in immobilizations in the range of 110–270 RU. For sample analysis, 7-point, 3-fold serial dilutions of aHSA, aHSA-HAP, aMSA and aMSA-HAP peptides (1000 nM to 15.5 nM) were prepared in HBS-EP+ buffer. For each injection, a flow rate of 40 µL/minute, contact time 300 s and dissociation time 2700 s was used. Between injections, the surface was regenerated with sequential injections of 10 mM glycine-HCl pH 1.5 with a flow rate of 30 µL/minute and contact time 30s. The data was fitted with the reference surface subtracted using a Biacore evaluation software 1:1 binding model.

*Peptide competition assay*: IL-17RA 2 µg/mL was immobilized on a CM5 sensor chip by amine coupling, typically resulting in immobilizations in the range of 110–270 RU. Binding of IL-17A (30 nM) was tested +/- aHSA-HAP or aMSA-HAP (250nM) in HBS-EP+ buffer. For each injection, a flow rate of 40 µL/minute, contact time 300 s and dissociation time 2700 s was used.

### *In-vivo* pharmacokinetics

FabT-aHSA and FabT-aMSA were dosed to BALB/c mice (*n=3*) via intravenous injection through the tail vein at 2 mg/kg. 25 μL blood samples were collected on K2 EDTA at 15 minutes, 30 minutes post-dose, followed by 1, 2, 4, 8, 24, 45, 96, 168 hours via the opposite tail vein. Blood was left to coagulate on wet ice for 30 minutes. 10-15 μL serum samples were stored at -80°C until analysis. Pharmacokinetic parameters were calculated in Phoenix 64 v8.3.4 (Certara, Princeton, NJ, USA) on the individual concentration/time profiles. Mean ± standard deviations were reported.

### Bioanalysis

Samples were prepared for analysis using a serum total lysis assay, where whole serum was lysed and the FabT-aHSA and FabT-aMSa concentrations were tracked using a signature peptide (VDNALQSGNSQESVTEQDSK), which was present in the human, but not the murine, Fab domain. Briefly, 5 µL of each serum sample were aliquoted in 96-well plate. Calibration standards, ranging from 1-500 µg/mL, and quality control samples were both prepared from FabT-aHSA and FabT-aMSa, and aliquoted in 96-well plate at 5 µL/well. An internal standard working solution was prepared using an L-Lysine-2HCl, 13C6, 15N2 labelled signature peptide (VDNALQSGNSQESVTEQDSK [underlining denotes labelled residue]), diluted in 33/67 0.1M Ammonium Bicarbonate/ACN, and added to each well of the plate. Samples were then denatured using 7 µL of TCEP (0.1 M final concentration) and incubated for 30 minutes at room temperature. Samples were then alkylated using 7 µL of iodoacetamide (0.2 M final assay concentration) and incubated protected from light for 30 minutes at room temperature. A mix of 7 µL of L-cysteine (0.1 M, Sigma #14495), 153 µL of 0.1 M ammonium bicarbonate buffer and 10 µL of trypsin (0.5 mg/mL, Sigma #T6567) in acetic acid was prepared. 170 µL of this mix were added to each well. Samples were incubated overnight for 16 to 21 hours at 37°C. Samples were then centrifuged for 5 minutes at approximately 1500 g. The trypsinization reaction was stopped by transferring 100 µL of supernatant to a 96-well plate containing 100 µL of 92% H2O 5% MeOH 3% formic acid.

The plate was analysed by LC-MS/MS. The instrument used was an ultra-performance liquid chromatography from Shimadzu coupled to a triple quadrupole mass spectrometer from Sciex (6500+ system). Stationary phase used was an ACE C4 column of 2.1x100 mm, 2.0 µm dimensions and mobile phases used were 5/95 ACN/H2O + 0.1% acetic acid (solvent A) and 95/5 ACN/H2O + 0.1% acetic acid (solvent B) at a flow rate of 0.5mL/min. A gradient, starting from 0% increasing to 10% of solvent B over 5min, was used to separate the analytes. The MS instrument was used in MRM mode and following transitions were used to monitor the two peptides of interest: 712.9->748.5 and 715.4->752.4 respectively for the signature peptide (VDNALQSGNSQESVTEQDSK) and the internal standard (VDNALQSGNSQESVTEQDSK [underlining denotes labelled residue]). Data processing was performed on Analyst software (Sciex).

### TNF reporter assay using HEK-Blue™ readout

The human CD40L reporter HEK 293 (HEK-Blue CD40L) cell line (*In vivo*Gen, #hkb-cd40) was used to determine TNFα activity in all assays. Stimulation of HEK-Blue CD40L cells by TNFα leads to downstream activation of the NF-κB pathway and production of secreted embryonic alkaline phosphatase (SEAP). Fabs were titrated on the assay plate (Sigma-Aldrich, #M3061) and preincubated with 10 pM of human TNFα (UniProt P01375) in the presence or absence of human serum albumin (HSA) (Sigma-Aldrich, #A1653) for 2 h at 37°C. HEK-Blue CD40L cells were added (10,000 cells/well) to the stimulus mixture and further incubated for 18 h (37°C, 5% CO_2_). The addition of HEK-Blue Detection medium (*In vivo*gen, #hb-det) was used for SEAP detection. Absorbance at 630 nm (BioTek, Synergy Neo 2) was measured following a 2 h incubation to determine SEAP levels. Percentage activities reported were calculated between media-only control and expected maximum activities within assays. The respective IC_50_ values were determined using 4PL fitted curves (GraphPad Prism 9).

### Crystallography and structure determination

Conditions suitable for crystal growth were identified by the sitting drop vapour diffusion method using commercially available crystallisation screens (Qiagen, Manchester, UK). To generate diffraction quality crystals, hanging drop vapour diffusion method was used, in which 1 μL of protein solution was mixed with 1 μL of reservoir solution. The reservoir contained either 3 M malic acid, pH 7.0, and 17% (v/v) polyethylene glycol 3350 for FabT or 0.2 M lithium chloride and 20% (v/v) polyethylene glycol 3350 for FabT-aHSA. Crystals were harvested, briefly incubated in mother liquor supplemented with 20% (v/v) glycerol and flash frozen in liquid nitrogen.

Datasets were collected from single crystals of FabT and FabT-aHSA at the Diamond Synchrotron (Oxfordshire, UK). For Sulfur-SAD (single-wavelength anomalous diffraction) phasing of FabT-aHSA, a dataset was collected from a second crystal at a wavelength of 1.6531 Å. The datasets were processed using XDS (33). The structure of FabT was solved by molecular replacement with Phaser (34) using the coordinates of an in-house Fab. Similarly, FabT-aHSA was solved using the coordinates of the final refined structure of FabT. Both structures were built and refined iteratively with Phenix (35) and COOT (36). Model geometry was validated using Molprobity (37).

To confirm the location of the cysteine residues in FabT-aHSA, molecular replacement using Phaser from CCP4i2 (38), was run with the final refined FabT-aHSA structure and the 1.6531 Å dataset. This was followed by REFMAC5 (39) to generate difference anomalous map coefficients. The latter was converted into an anomalous difference map using Phenix tool, mtz2map. Molecular visualisations were generated with Pymol (The PyMOL Molecular Graphics System, Version 2.0 Schrödinger, LLC) (39) and MOE (Molecular Operating Environment). Data collection and refinement statistics are summarised in **Table S1**.

### Epitope mapping by hydrogen-deuterium exchange mass spectroscopy

For HDX-MS analysis, 20 µM of HSA was complexed with 200 µM of aHSA peptide and 20 µM of MSA was complexed with 200 µM of aMSA peptide and incubated for 1 hour at 4°C. 4 μL of each serum albumin alone or complexed with the peptides were diluted into 57 μL of 10 mM phosphate in H_2_O (pH 7.0), or into 10 mM phosphate in D_2_O (pD 7.0). Deuterium labelling was performed for several time points: 30 s, 2 min, 15 min and 1 h) at 20°C. After the reaction, all samples were quenched by mixing at 1:1 with a 100 mM phosphate buffer containing 4 M Guanidine Hydrochloride and 250 mM TCEP at 1°C (final pH read 2.4). The mixture was immediately injected into the nanoAcquity HDX module (Waters Corp.) for peptic digest. Peptide digestion was then performed on-line using an online Enzymatic pepsin digestion column (Waters) in 0.2% formic acid in water at 20°C and with a flow rate of 100 μL/min. The peptic fragments were then trapped onto an Acquity BEH C18 1.7 μM VANGUARD chilled pre-column (Waters) for 3 min. Peptides were eluted into a chilled Acquity UPLC BEH C18 analytical column (1.7 μM 1.0 × 100, Waters) with a linear gradient raising from 8 to 40% of solvent B (0.2% formic acid in acetonitrile) at a flow rate of 40 μL/min and at 0°C. To prevent significant peptide carryover, the protease column was washed three times between runs with pepsin wash (0.8% formic acid, 1.5 Gu-HCl, 4% MeOH) and a blank run was performed between each sample. All deuterated time points and un-deuterated controls were carried out in triplicate with blanks run between each data-point. The eluted peptide fragments were ionized by positive electrospray into a Synapt G2-Si mass spectrometer (Waters). Data acquisition was run in ToF-only mode over an m/z range of 50-2000 Then, using an MSe method (low collision energy, 4 V; high collision energy: ramp from 18 V to 40 V). Glu-1-Fibrinopeptide B peptide was used for lock mass correction. The mass spectrometer was calibrated with sodium iodide.

MSE data from un-deuterated controls samples were used for sequence identification using the Waters Protein Lynx Global Server 2.5.1 (PLGS). Ion accounting files for the 3 control samples were combined into a peptide list imported into DynamX (v3.0). Peptides were filtered in DynamX using these parameters: were a minimum and maximum peptide sequence length of 4 and 25, respectively, minimum intensity of 1000, minimum MS/MS products of 2, minimum products per amino acid of 0.2, and a maximum MH+ error threshold of 10 ppm. DynamX v3.0 was used to quantify the isotopic envelopes resulting from deuterium uptake for each peptide at each time-point. All the spectra were examined and checked visually to ensure correct assignment of m/z peaks and only peptides with a high signal to noise ratios were used for HDX-MS analysis. Statistical analysis was performed with Deuteros software (40) applying a 99% confidence interval.

### Domain mapping

*Serum albumin mutant generation and expression*: Genes for expression of serum albumin mutants were synthesised and cloned into a human Fc vector for generation of C-terminal Fc tagged constructs. Constructs were subsequently transfected into Expi293F™ Cells using the ExpiFectamine™ 293 Transfection Kit according to the manufacturer’s instructions. After one week incubation the cells were isolated by centrifugation and the supernatant recovered. Supernatants were diluted 1:2 in binding buffer (Thermo Scientific™ Fish Serum Blocking Buffer (1:10 dilution), PBS, 0.05% Tween20, 0.025% sodium azide) and stored at 4°C until use.

*Serum albumin-Fc binding by Octet:* Binding experiments for epitope determination were carried out on an Octet HTX instrument. Anti-hIgG Fc Capture (AHC) Biosensors were soaked in binding buffer for 600s, followed by loading of serum albumin-Fc mutants for 300s. After dissociation for 180s in binding buffer, association was measured in 10 µg/mL of either FabT-aHSA or FabT-aMSA for 600s, followed by dissociation in binding buffer for 900s. Data was analysed in Octet Software ‘Data Analysis 10.0’. The maximum load, maximum association and maximum dissociation values were extracted for all biosensors. The impact on association and dissociation of introducing surface mutations at different positions was then assessed, with maximum loading acting as a normalisation factor for poorly expressing mutants.

### Solid-phase peptide synthesis of knob domain – HAP chimeric peptides

Detailed solid-phase peptide synthesis protocols and accompanying QC are displayed in the **Supplementary Materials (S8)**.

### Inhibition of IL-17 induced IL-6 release from dermal fibroblasts

Neonatal human dermal fibroblasts (106-05n, Sigma-Aldrich, Missouri, USA) were diluted in growth media, DMEM (21969-035, Invitrogen) with 10% (*v/v*) foetal calf serum (10082, Invitrogen) and 2mM L-glutamine (25030, Invitrogen), plated in 384-well tissue culture treated plates (3701, Corning) at 3.125x10^4^ cells/ml, 40 µL per well and incubated at 37°C, 5% CO_2_ for three hours. Test articles were diluted in vehicle (DMSO, 23500.260, VWR) to give a final top concentration of 3 µM (DMSO final 0.5% *v/v*) and serially diluted to provide a 20-point, 2.5-dilution curve. The ligands TNFα and IL-17A, produced at UCB, were diluted together in growth media to give concentrations of 169 pM and 333.3 pM, respectively. Test articles, or vehicle control and ligands were combined and incubated for five hours at 37°C, 5% CO_2_ before being added to the human dermal fibroblasts. Final concentrations of TNFα and IL-17A on the cells were 25 pM and 50 pM, respectively. Cells were incubated with test articles and ligands for 18 ± 2 hours at 37°C, 5% CO2. Interleukin 6 (IL-6) levels were measured using a human IL-6 HTRF kit (62HIL06PEH, Cisbio). Briefly, 50 µL each of europium cryptate and Alexa 665 were combined with 4.5 ml of detection buffer, and 10 µL added per well to a 384-well, low volume, white plate (Greiner, 784075). Cell free supernatant was diluted 1:3 in growth media and 10 µL added to the HTRF assay plate, followed by two hours incubation at room temperature in the dark, with shaking at 300 RPM. Fluorescence in all wells was measured using the Synergy Neo 2 microplate reader with excitation at 330 nm and emission at 620 and 665 nm. The ratio between fluorescence emissions at 665 and 620 nm were used to calculate the percent inhibition of IL-6 by the test articles compared with vehicle control cells stimulated with TNFα and IL-17A, or IL-17A alone.

### Accession codes

Structure factors and coordinates have been deposited in the PDB (PDB accession codes: 8C7V and 8C7J).

## Data availability statement

The original contributions presented in the study are included in the article/**Supplementary Material**. Further inquiries can be directed to the corresponding authors.

## Ethics statement

The animal study was reviewed and approved by University of Reading Animal Welfare Ethical Review Body approval (Personal Project Licence: 70/8108 “Antibody Production”). 

## Author contributions

The study was conceived and supervised by AM, RA, AS-T, AL, JE and RP. Reagents were generated by RA, KH and CM. Data were generated by RA, CJ, MK, KH, ZA, MB, KC, and EG. Analysis was performed by JS, KM and RA. The manuscript was written by AM, RA, CJ and MK with input from all authors. All authors contributed to the article and approved the submitted version. 
