# Supplementary material for: Serum albumin binding knob domains engineered within a VH framework III bispecific antibody format and as chimeric peptides
Source: Front Immunol. 2023 May 12;14:1170357. doi: 10.3389/fimmu.2023.1170357 (PMC10213618; doi:10.3389/fimmu.2023.1170357)
Supplement: Supplementary file 1 [file DataSheet_1.docx]

**Supplementary Material**

**Serum albumin binding knob domains engineered within a V_H_ framework III bispecific antibody format and as chimeric peptides**

Ralph Adams^1$^, Callum Joyce^1$^, Mikhail Kuravskiy^1$^, Katriona Harrison^2,3^, Zainab Ahdash^1^, Matthew Balmforth^1^, Kelda Chia^1^, Cinzia Marceddu^1^, Matthew Coates^1^, James Snowden^1^, Emmanuel Goursaud^4^, Karelle Ménochet^1^, Jean van den Elsen^5^, Richard J. Payne^2,3^, Alastair D. G. Lawson^1^, Anthony Scott-Tucker^1^, Alex Macpherson^1^*^#^

^1^ UCB Biopharma UK, Slough, UK.

^2^ School of Chemistry, The University of Sydney, Sydney, NSW 2006 Australia.

^3^ Australian Research Council Centre of Excellence for Innovations in Peptide and Protein Science, The University of Sydney, Sydney, NSW 2006 Australia.

^4^ UCB Biopharma SA, Braine L’Alleud, Belgium.

^5^ University of Bath, Bath, UK.

^$^ These authors contributed equally to this work and share first authorship.

* [alexander.macpherson@lilly.com](mailto:alexander.macpherson@lilly.com)

^#^ Present address: Eli Lilly and Company, Bracknell, UK.

**S1 Table 1 for structure collection and refinement.**

|  | **FabT-aHSA** | **FabT** |
| --- | --- | --- |
| **Wavelength** | 0.9763 | 0.9795 |
| **Resolution range** | 62.9 - 2.0 (2.07 - 2.0) | 63.9 - 1.61 (1.65 - 1.61) |
| **Space group** | P 2_1_ 2_1_ 2_1_ | P 1 2_1_ 1 |
| **Cell dimensions** |  |  |
| **a, b, c (Å)** | 63.24, 71.31, 125.89 | 60.6, 127.93, 93.81 |
| **α, β, γ (^o^)** | 90.00, 90.00, 90.00 | 90.00, 95.47, 90.00 |
| **Total reflections** | 492364 (33371) | 585312 (29677) |
| **Unique reflections** | 39199 (3837) | 178845 (11053) |
| **Multiplicity** | 12.6 (8.7) | 3.3 (2.7) |
| **Completeness (%)** | 99.90 (99.12) | 97.5 (81.5) |
| **I/σI** | 19.71 (4.43) | 17.0 (2.2) |
| **R-merge** | 0.07166 (0.441) | 0.034 (0.418) |
| **CC1/2** | 0.999 (0.942) | 0.999 (0.655) |
| **Reflections used in refinement** | 39196 (3838) | 178842 (15350) |
| **Reflections used for R-free** | 1944 (204) | 8952 (772) |
| **R-work** | 0.1859 (0.2194) | 0.1662 (0.3188) |
| **R-free** | 0.2257 (0.2635) | 0.1943 (0.3417) |
| **Number of non-hydrogen atoms** | 4094 | 11498 |
| **macromolecules** | 3691 | 9969 |
| **ions** | 5 | 0 |
| **solvent** | 398 | 1529 |
| **R.m.s. deviations** |  |  |
| **bond lengths (Å)** | 0.01 | 0.01 |
| **bond angles (^o^)** | 1.07 | 1.06 |
| **Ramachandran favoured (%)** | 95.82 | 98.38 |
| **Ramachandran allowed (%)** | 3.56 | 1.62 |
| **Ramachandran outliers (%)** | 0.63 | 0 |
| **Average B-factor** | 47.98 | 24.21 |
| **macromolecules** | 48.07 | 23.10 |
| **ions** | 54.80 | 0 |
| **solvent** | 47.08 | 31.44 |

**S2 Sulphur-SAD map confirming locations of disulphide bonds in aHSA knob domain.**

Peaks of the anomalous difference map of FabT-aHSA determined by sulphur-SAD, contoured at 3.0σ above mean, are shown in dark blue. Heavy chain is shown in cyan, light chain in green, cysteines and methionine residues as sticks, and chloride (CL) atoms in orange. (A) knob domain with 8 cysteine residues forming 4 disulphide bonds (B) Heavy and light interchain disulphide bond (C) Variable heavy chain domain showing intrachain disulphide bond and 2 methionine residues (D) Variable light chain domain showing intrachain disulphide bond and 1 methionine residue (E) Constant heavy chain domain 1 showing intrachain disulphide bond (F) Constant light chain domain showing intrachain disulphide bond


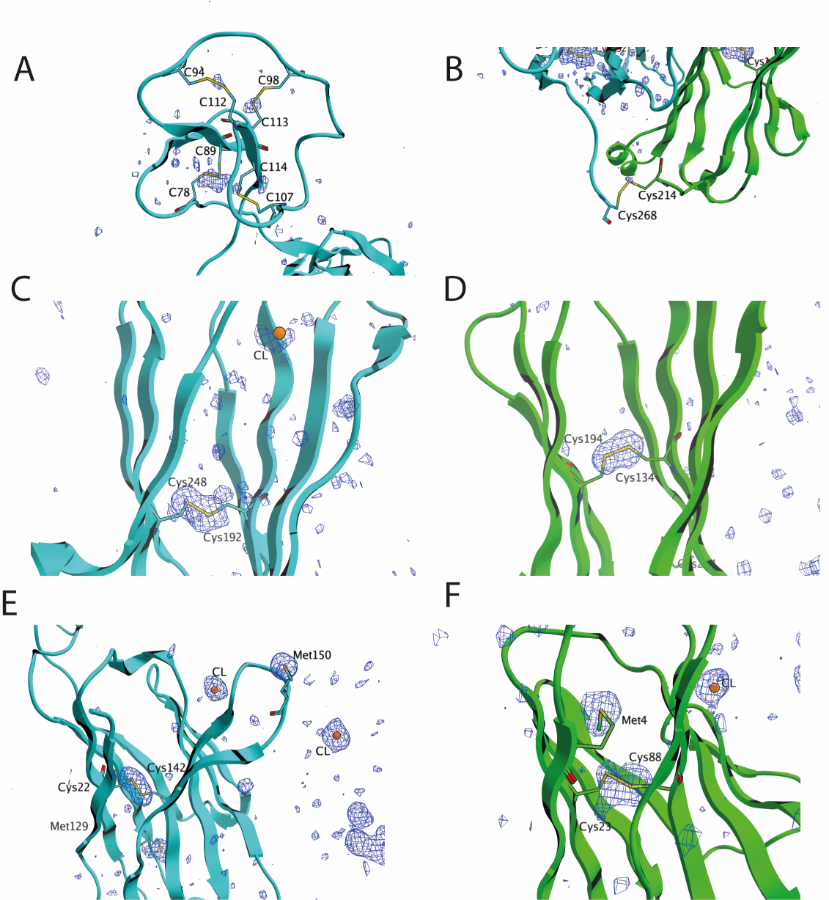


**S3 Electron density map of FabT-aHSA.**

2Fo-Fc electron-density map contoured at 1.0σ above mean, is shown in dark blue. Heavy chain is shown in cyan, light chain in green. (A) FabT-aHSA (B) aHSA knob domain


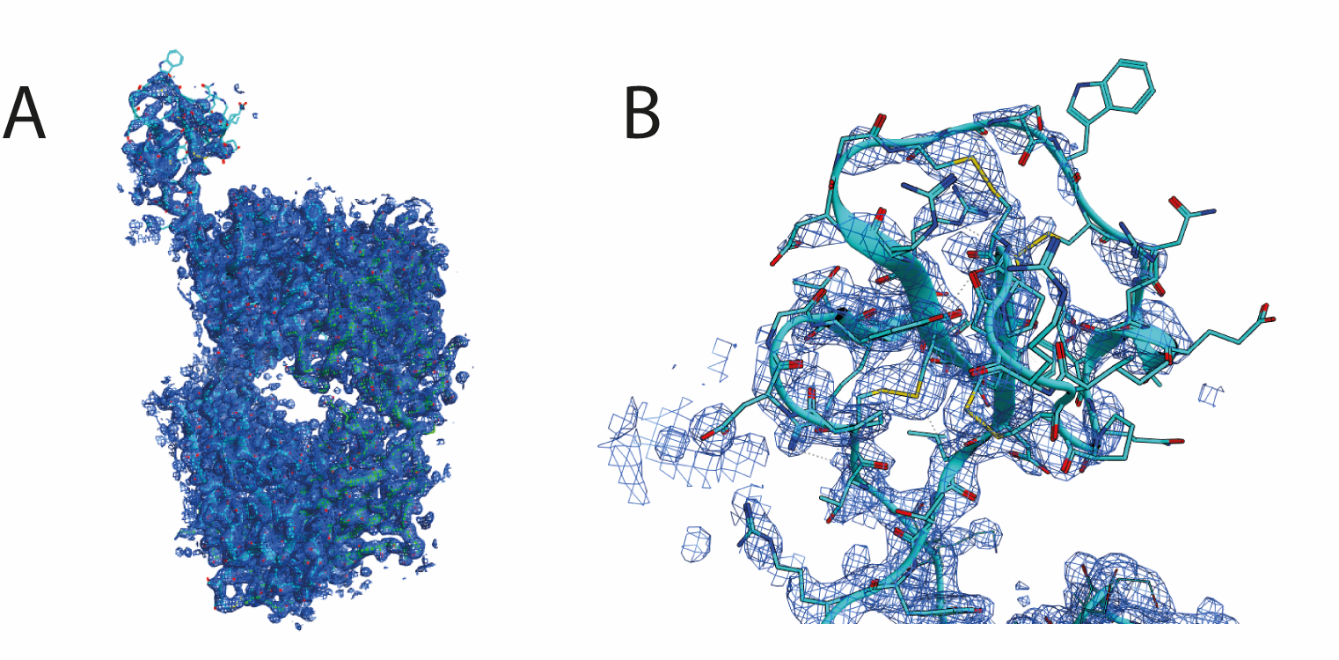


**S4 Peptide coverage map and time course data for HDX**

Panel A shows peptide coverage for HSA. Panel B show Woods plots for all experimental timepoints. Blue and red regions indicate a negative (protected) and a positive (deprotected) deuterium uptake difference, respectively.


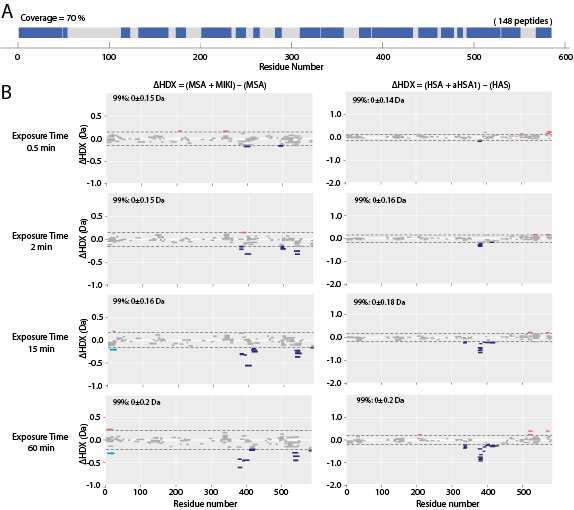


**S5 Amino acid mutations for HSA and effects on FabT-aHSA association and dissociation rates, as determined by Octet bilayer interferometry**

| **HSA mutation** | **Load** | **Association** | **Dissociation** |
| --- | --- | --- | --- |
| Wild type | 2.1127 | 1.239 | -0.1678 |
| A-323-K_A-324-D | 1.8407 | 0.2326 | -0.091 |
| A-323-K_A-327-L_A-354-E | 2.1915 | 0.2988 | -0.0779 |
| A-325-V_A-329-M | 1.854 | 1.4527 | -0.1928 |
| A-331-L_A-343-V | 2.1913 | 1.3611 | -0.1659 |
| A-338-H_A-340-D | 1.8159 | 0.2436 | -0.0657 |
| A-341-Y_A-381-V | 2.3186 | 0.7853 | -0.4135 |
| A-374-F_A-375-D | 2.0602 | 1.135 | -0.4061 |
| A-375-D_A-378-K | 1.8549 | 0.5464 | -0.1753 |
| A-376-E_A-379-P | 2.5103 | -0.0104 | -0.0051 |
| A-380-L_A-381-V | 2.0494 | 0.0145 | -0.0054 |
| A-382-E_A-385-Q_A-386-N | 2.0369 | 1.0062 | -0.2299 |
| A-383-E | 1.7687 | 0.0346 | 0.0023 |
| A-387-L | 1.7213 | 0.366 | -0.1025 |
| A-389-K_A-393-E | 2.2848 | 1.2924 | -0.1996 |
| A-390-Q_A-391-N | 2.2467 | 0.9909 | -0.1877 |
| A-396-E | 1.6021 | 0.7345 | -0.1031 |
| A-397-Q_A-398-L | 1.488 | 0.8643 | -0.1012 |
| A-401-Y_A-402-K | 2.0733 | 1.1366 | -0.1684 |
| A-417-Q_A-496-T | 1.5902 | 0.3998 | -0.0765 |
| A-421-P_A-527-T | 1.8786 | 0.9934 | -0.1603 |
| A-425-E_A-429-N | 1.866 | 0.9503 | -0.153 |

**S6** **Amino acid mutations for MSA and effects on FabT-aMSA association and dissociation rates, as determined by Octet bilayer interferometry**

| **MSA mutation** | **Load** | **Association** | **Dissociation** |
| --- | --- | --- | --- |
| Wild type | 3.1308 | 1.2171 | -0.1762 |
| A-389-K | 2.1392 | 0.2482 | -0.0534 |
| A-417-Q | 2.5609 | 0.9064 | -0.1196 |
| A-547-V_A-550-D | 2.6478 | 1.283 | -0.1621 |
| A-375-A_A-378-Q | 2.4301 | 1.0511 | -0.1456 |
| A-390-T_A-391-N | 2.289 | -0.0293 | -0.0688 |
| A-577-T_A-578-R | 2.5567 | 0.9287 | -0.1392 |
| A-376-E_A-379-P | 2.7285 | 1.2378 | -0.1858 |
| A-394-L_A-397-K_A-398-L | 2.0559 | -0.0204 | -0.0253 |
| A-421-P | 2.5344 | 1.1424 | -0.1666 |
| A-580-K_A-581-D | 2.5768 | 1.0869 | -0.1453 |
| A-380-L_A-381-V | 2.3777 | 0.4609 | -0.1788 |
| A-395-Y_A-396-E | 2.1275 | 0.1085 | -0.0265 |
| A-425-E_A-410-R | 2.2835 | 0.658 | -0.1157 |
| A-382-E_A-385-K_A-386-N | 2.2579 | 0.6557 | -0.305 |
| A-532-L_A-536-K_A-547-V | 2.1042 | 0.1674 | -0.0449 |
| A-383-E | 2.208 | -0.0303 | -0.0341 |
| A-401-Y_A-402-G | 2.3438 | 0.2024 | -0.0423 |
| A-536-K_A-537-P | 2.1874 | 0.0541 | -0.0294 |
| A-387-L | 2.2861 | 0.0112 | -0.0826 |
| A-540-T_A-541-A_A-543-Q | 2.53 | 0.8373 | -0.1289 |
| A-389-K_A-393-D | 2.6957 | 1.2764 | -0.1488 |
| A-409-V_A-413-Q_A-538-K | 2.3263 | 0.3146 | -0.2097 |
| A-542-E_A-545-K_A-549-D | 2.702 | 1.2173 | -0.148 |

**S7 Detailed procedures for phage display**

**Harvesting of immune material and ELISA assay**

Seven days following the second and third immunisations, serum samples were taken to assess target-specific responses using ELISA. Protein was adsorbed directly to Nunc Maxisorb plates at 2 µg/ml in PBS. Serum samples were diluted into PBS supplemented with 1 % BSA (w/v), and the response determined using a secondary antibody anti-Bovine H+L – HRP conjugate (Stratech). After confirmation of a selective serum titre response, ten days after final boost additional sampling was taken from these cows: 500 mL blood, a sample of spleen, ~ 2 cm^3^ and a single draining lymph node taken proximal to the site of immunisation.

**Total RNA extraction and RT PCR**

An RNeasy plus midi kit (Qiagen) was used to purify total RNA, as per the manufacturer’s protocol. Briefly, 5x10^7^ lymph node cells were pelleted by centrifugation. The pellet was resuspended with RLT buffer containing 1 % β-mercaptoethanol (v/v) and the solution was homogenised using a Tissueruptor 2 (Qiagen). The homogenate was diluted 1:1 with 70 % ethanol and applied to RNeasy column. The column was washed once with buffer RW1 and twice with RPE buffer. RNA was eluted from the column using nuclease free water.

RNA was immediately used in a RT-PCR reaction using Super script IV vilo Master Mix (Invitrogen). A 40 µL reaction was set up using 0.8 µg of RNA per reaction, 8 µL of Super script IV vilo Master Mix and nuclease free water to bring each reaction volume to 40 µL. The reaction was placed into a thermocycler at 25 °C for 10 minutes, 50 °C for 10 minutes and 85 °C for 5 minutes.

**Primary PCR**

The CDRH3 regions were selectively amplified via PCR using primers annealing to the framework-3 and -4 of the heavy chain, thereby amplifying the CDRH3 region regardless of length (standard or ultralong CDRH3). The primers used (from 5’ to 3’) were:

Forward primer: GGACTCGGCCACMTAYTACTG

Reverse primer: GCTCGAGACGGTGAYCAG

For the PCR, a 50 µL PCR reaction was set up comprising: 25 µL Phusion Green Hotstart II Master mix (Thermo scientific); 2.5 µL of the forward and reverse primers (both at 10 µM), 2 µL of RT PCR product and 25 µL Nuclease-free water and 2.5 µL DMSO.

The reaction mixture was heated in a thermocycler as follows: 98 °C for 30 seconds, followed by thirty cycles of (98 °C for 10 seconds; 62 °C for 30 seconds; 72 °C for 30 seconds) and a final extension of 72 °C for 5 minutes.

Primary PCR DNA was column purified (QIAquick PCR purification kit) before being used in a secondary PCR.

**Secondary PCR**

Primer sets derived from ultralong CDRH3 ascending and descending stalk sequences were used to specifically amplify ultralong sequences from the primary PCR DNA. 7 ascending stalk primers were used individually in sperate reactions whilst 6 descending stalk primers were pooled. Ascending primers were used at a concentration of 10 µM and each descending primer was at a concentration of 10 µM in the pooled solution. Primer sets also contained *SfiI* and *NotI* restriction enzyme sites which allowed for ligation into vector.

The primers used (from 5’ to 3’) were as follows:

**Ascending primer set**

CTCGCGGCCCAGCCGGCCATGGCCACTACTGTGCACCAAAAAACA

CTCGCGGCCCAGCCGGCCATGGCCACTACTGTGCACCAAAGAACC

CTCGCGGCCCAGCCGGCCATGGCCACTACTGTGCACCAAAAAACG

CTCGCGGCCCAGCCGGCCATGGCCACTACTGTGCACCAACAAACT

CTCGCGGCCCAGCCGGCCATGGCCACTACTGTGCACCAACAGACC

CTCGCGGCCCAGCCGGCCATGGCCACTACTGTGGTCCAGAAAACA

CTCGCGGCCCAGCCGGCCATGGCCACTACTGTAGTCCAACGAACA

**Descending primer set**

TGATGGGCGGCCGCGGCATCGACGTACCATTCGTA

TGATGGGCGGCCGCGGTATCGACGTACCATTCGTA

TGATGGGCGGCCGCGGCTTCGACGTACAATTCGTA

TGATGGGCGGCCGCGGCATTGACGTAGAATTCGTA

TGATGGGCGGCCGCGGCCTCGATGTCAAATTCGTA

TGATGGGCGGCCGCGGTTTCGACGTGGTATTCGTA

A 50 µL PCR was set up comprising: 25 µL Phusion Green Hotstart II Master mix (Thermo scientific); 2.5 µL of the forward primer at 10 µM and 2.5 µL of the pooled reverse primer with each at 10 µM; 2 µL (50ng total template DNA) of primary PCR product; 25 µL Nuclease free water and 2.5 µL DMSO.

The reaction mixture was heated in a thermocycler as follows: 98 °C for 30 seconds, followed by thirty cycles of: (98 °C for 10 seconds; 62 °C for 30 seconds; 72 °C for 30 seconds) and a final extension of 72 °C for 5 minutes. The secondary PCR product was column purified before being used for sub-cloning into the phagemid vector.

**Library Construction**

A phagemid vector (derivative of pUC119, Hoogenboom *et al*. (1991) Nucleic Acids Res. 11;19(15): 4133-7) was used throughout research. Both the phagemid vector and the secondary PCR product were digested using *SfiI* (7.5 U/µg DNA) at 50 °C for 4 h followed by *NotI* (10 U/µg DNA) at 37 °C overnight. Products were gel-extracted (Qiagen gel extraction kit) prior to ligation. Vector and ultralong CDRH3 inserts were ligated using a 1:3 molar ratio. Ethanol precipitation was used to increase concentration, purity and remove salt from ligation prior to transformation into *E. coli*.

The purified ligation reaction was electroporated into *E. coli* TG1 (Lucigen) with ~ 0.5 µg vector DNA per cuvette. 1 mm Micropulser electroporation cuvettes (Biorad) were shocked at 10 µF, 600 Ω and 1,800 V. 1 mL of pre-warmed recovery medium (provided with TG1 cells) was added to each cuvette after shocking to resuspend the cells, within 10 seconds of the pulse. Electroporated cells were pooled for recovery at 37 °C for 1 hour with shaking at 225 rpm. Recovered samples were plated out onto 2TY Agar + 1 % Glucose + 100 µg/mL Carbenicillin and incubated at 30 °C overnight.

**Phage Rescue**

The solid phase culture biomass was harvested by addition of 2TY media + 1% Glucose + 100µg/mL Carbenicillin to the plates and scraping into liquid culture to resuspend. The collected culture was used to seed fresh 2TY media + 1 % Glucose + 100 µg/mL Carbenicillin at an OD600 of 0.1 AU. The culture was incubated at 37 °C with shaking at 225 rpm until it reached an approximate OD600 of 0.5 AU. M13K07 helper phage was added at a multiplicity of infection (MOI) of 20 and the culture was left standing at 37 °C for 1 hour. The culture was then centrifuged, and the pellet resuspended in 2TY media + 50 µg/mL Kanamycin + 100 µg/mL Carbenicillin (and no glucose) and incubated at 30 °C with shaking at 225 rpm overnight.

Following overnight incubation, the culture supernatant was recovered by centrifugation at 6,000 x g and mixed 5:1 with a solution of 20 % PEG-8000, 2.5M NaCl and incubated on ice for 1 hour. Following centrifugation at 10,000 x g, the phage pellet was resuspended in 20 mL of PBS. A further round of precipitation was performed, and the phage pellet resuspended in PBS supplemented with 20 % glycerol at a final concentration of approximately 10^12^ PFU/mL. Purified phage aliquots were stored at -80 °C until required.

**Phage Bio-Panning**

Two rounds of enrichment were performed with human and mouse serum albumin. 1 x 10^12^ phage were blocked by mixing 1:1 with 2 % milk powder (w/v) in PBS for 30 minutes. Biotinylated antigen was added to the blocked phage at a concentration of 100 nM and incubated at room temperature with end over end mixing for 1 hour.

50 µl Streptavidin Dynabeads (Thermofisher) were washed once with 1ml of PBS and a further 3 times using 1 mL 2 % milk powder (w/v) block solution. The beads were then resuspended in 1 mL of 2 % milk powder and incubated at room temperature for 1 hour with end over end mixing. After incubation the streptavidin beads were pelleted, and the supernatant removed. The beads were then resuspended in the blocked phage solution, incubated for 10 minutes with mixing, and washed four times with 1 mL of 0.1% Tween 20 in PBS.

After washing, beads were pelleted, and supernatant removed. 500 µL of 0.1 M hydrochloric acid was added to elute bound phage from beads. After 5 minutes of incubation, 500 µL of 1 M Tris-HCl was added to neutralise the acid. Phage solution was added to 10 mL of mid-log growing *E. coli* TG1 cells in 2TY media and incubated at 37 °C for 30 minutes without shaking to allow for bacterial infection.

A 5-point 10-fold serial dilution of infected cells into 2TY was preformed and 50 µL of each dilution was plated on small titres plates containing 2TY Agar + 100 µg/mL Carbenicillin. Titre plates were incubated overnight at 37°C. The remaining culture was centrifuged at 3,000 x g for 10 minutes. The pellet was resuspended in 1 mL of 2TY and spread across a 25cm x 25cm petri dish of 2TY Agar + 100 µg/mL Carbenicillin and incubated overnight at 30°C. A second round of enrichment was achieved by repeating the phage rescue and bio-panning procedures above.

**Monoclonal Phage Screening ELISA**

Colonies were picked from output titres plates and placed into a 96-well culture plate containing 1 mL of 2TY media + 1% Glucose + 100 µg/mL Carbenicillin and left shaking overnight at 37°C.

The following morning, 100 µL of the overnight cultures was transferred to a fresh 96-well culture block containing 1 mL of 2TY media + 1% Glucose + 100µg/ml Carbenicillin and was incubated at 37°C until wells reached an approximate O.D600 of 0.5. To this culture, 50 µL of M13K07 helper phage diluted in 2TY to give a final MOI of 20 was added to each well. The block was incubated at 37°C without shaking for 1 hour and then centrifuged at 2,000 x g. Pelleted cells were resuspended in 1 mL of 2TY media supplemented with 50 µg/ml Kanamycin and 100 µg/ml Carbenicillin. The block was then incubated overnight at 30 °C with shaking.

Binding to antigen was assessed by monoclonal phage ELISA, 96-well flat bottom Nunc MaxiSorp plates (Thermofisher) were coated with a 2.5 µg/ml solution of human, mouse, or rat serum albumin and left overnight at 4 °C. A negative control plate and Anti-myc tag plate to check display were also prepared.

Enriched monoclonal rescue blocks were centrifuged at 2,000 x g and 500 µl of supernatant was moved to a fresh block containing an equal volume of a 2 % milk (w/v) in PBS to block phage. The coating solution was removed from coated Nunc plates and each plate was blocked with 300 µl 1 % milk powder. Both phage and Nunc plates were blocked for 1 hour at room temperature. Nunc plates were washed using a 96-well microplate washer (BioTek) with a PBS solution contain 0.1% Tween20 (Sigma Aldrich). Excess wash solution was removed by tapping plates against a paper towel. 100 µL of blocked phage solution was added to each well of Nunc MaxiSorp plate and left shaking at room temperature for 1 hour. Plates were washed and dried as previously described. 100 µL of an Anti-M13-Horse radish peroxidase conjugated antibody (GE Healthcare) diluted (1:5,000) in a 1 % milk solution was added to each well and left shaking at room temperature for 1 hour. Plates were washed again and 50 µL of TMB solution (Merck Millipore) was added to each well. Plates were left shaking for 10 minutes. Absorbance was measured at 630 nm. Binding signals were obtained where Absorbance at 630 nm was > 3-times the signal upon irrelevant antigen.

For sequencing, 0.5 µL of cell containing media from enriched monoclonal rescue blocks was added to the corresponding wells in a PCR plate containing: 20.75 µL of DPEC-treated water; 2.5 µL of 10x Standard Taq buffer (New England Biolabs); 0.5 µL of forward and reverse 10 µM primer stock and 0.25 µL of Taq DNA polymerase (5000u/mL – New England Biolabs).

The primers used (from 5’ to 3’) were as follows:

Forward: GTTGGCCGATTCATTAATGCAG

Reverse: ACAGACAGCCCTCATAGTTAGC

The plate was heated to 95°C for five minutes in a thermocycler and then heated for thirty-five cycles of: (95 °C for 40 seconds; 55 °C for 40 seconds; 68 °C for 100 seconds) and a final extension of 72 °C for 2 minutes. Finally, 1 µL Illustra ExoProStar was added to each well to remove unused dNTP’s and primers before sequencing. The plate was placed in a thermocycler at 37 °C for 40 minutes and 80 °C for 15 minutes, prior to Sanger sequencing, performed at Macrogen.

**Display levels of Albumin phage library**

| **Library** | **Display %** |
| --- | --- |
| Unenriched albumin library | 89% (85/95 picked colonies) |
| HSA enriched Round 1 | 40% (38/95 picked colonies) |
| HSA enriched Round 2 | 97.9% (93/95 picked colonies) |
| MSA enriched Round 1 | 96.8% (92/95 picked colonies) |
| MSA enriched Round 2 | 85.3% (81/95 picked colonies) |

# S8 General Procedures for peptide synthesis

### Materials and Methods

Peptide grade *N,N*-dimethylformamide (DMF) for peptide synthesis was purchased from RCI Labscan. Gradient grade acetonitrile (MeCN) for chromatography was purchased from Sigma Aldrich and ultrapure water (Type 1) was obtained from a Merck Millipore Direct-Q 5 Water Purification System. Standard Fmoc-protected amino acids (Fmoc-Xaa-OH), coupling reagents and resins were purchased from Mimotopes or Novabiochem. Fmoc-SPPS was performed through automated synthesis on a Syro I peptide synthesizer (Biotage). All other reagents were purchased from Sigma Aldrich, AK Scientific or Merck and used as received.

**Fmoc-Solid-Phase Peptide Synthesis (SPPS)**

**General procedure A;** Loading to 2-Chlorotrityl Chloride Resin (2-CTC): 2-CTC resin (0.8 – 1.5 mmol/g, Mimotopes) was swollen in dry CH_2_Cl_2_ (5 mL) for 10 min and then washed thoroughly with CH_2_Cl_2_ (5 × 5 mL). A solution of 5 vol% iPr_2_NEt in dry CH_2_Cl_2_ (5 mL) was added and the resin was shaken for 1 h at 25 ^o^C and washed with CH_2_Cl_2_ (5 × 5 mL). The washed resin was then treated with a solution of the C-terminal Fmoc-Xaa-OH (4 eq.) and iPr_2_NEt (8 eq.) in dry CH_2_Cl_2_ (final concentration 0.2 M) was added to the resin and the mixture was agitated for 16 h. The resin was washed with CH_2_Cl_2_ (3 × 5 mL), DMF (3 × 5 mL) and CH_2_Cl_2_ (3 × 5 mL) and treat with a solution of CH_2_Cl_2_/MeOH/iPr_2_NEt (17:2:1 v/v/v, 3 mL) for 5 min at 25 ^o^C. The resin was finally washed with CH_2_Cl_2_ (3 × 5 mL), DMF (3 × 5 mL), CH_2_Cl_2_ (3 × 5 mL), DMF (3 × 5 mL) and subjected to loading estimation conditions, followed by iterative peptide assembly (Fmoc-SPPS).

**General procedure B;** Estimation of amino acid loading: A small portion of the resin was treated with 2 vol.% 1,8-Diazabicyclo[5.4.0]undec-7-ene in DMF (2 mL) and shaken for 30 min at 25 ^o^C. The solution was diluted into 8 mL MeCN. A 1 mL aliquot was diluted up to 12.5 mL with MeCN and the UV absorbance of the resulting solution was measured (λ = 304 nm) to estimate the amount of amino acid loaded onto the resin.

**General procedure C;** Automated Peptide Synthesis (SYRO I peptide synthesizer): The resin (90 mg, 50 µmol, 0.56 mmol g^-1^, 1 eq.) was treated with 40 vol.% piperidine in DMF (800 µL) for 4 min, drained, then treated with 20 vol.% piperidine in DMF (800 µL) for 4 min, drained, and washed with DMF (4 × 1.2 mL). The resin was then treated with a solution of Fmoc-Xaa-OH (200 µmol, 4 eq.) and Oxyma (220 µmol, 4.4 eq.) in DMF (400 µL), a 1 wt.% solution of 1,3-diisopropyl-2-thiourea in DMF (400 µL), followed by a solution of DIC (200 µmol, 4 eq.) in DMF (400 µL). Coupling reactions were conducted at 40 ^o^C for 15 min. The resin was then drained and washed with DMF (4 × 1.2 mL) before being treated with a solution of 5 vol.% Ac_2_O and 10 vol.% *i*Pr_2_NEt in DMF (800 µL) for 6 min at room temperature, drained and washed with DMF (4 × 1.2 mL). For N-terminal acetylation, after the final coupling the resin was treated with 40 vol.% piperidine in DMF (800 µL) for 4 min, drained, then treated with 20 vol.% piperidine in DMF (800 µL) for 4 min, drained, and washed with DMF (4 × 1.6 mL). The resin was then treated with a solution of 5 vol.% Ac_2_O and 10 vol.% *i*Pr_2_NEt in DMF (800 µL) for 6 min at rt, drained and washed with DMF (4 × 1.2 mL).

**General procedure D:** Manual cleavage: The resin was thoroughly washed with CH_2_Cl_2_ (5 × 5 mL) before being treated with 80:5:5:5:5 v/v/v/v/v TFA:tri*iso*propylsilane:thioanisole:1,2-ethanedithiol:H_2_O and shaken at room temperature for 2 h. The resin was filtered, and the filtrate concentrated under a stream of nitrogen before addition of diethyl ether (40 mL). The peptide was pelleted by centrifugation (4 min, 4 ^o^C, at 5000 rcf) and the ether was decanted. The crude peptide was dissolved in the minimum volume of 1:1 MeCN/H_2_O and concentrated by lyophilization.

### Preparative High-Performance Liquid Chromatography

Preparative and semi-preparative reversed-phase high performance liquid chromatography (HPLC) was performed using a Waters 600E multisolvent delivery system with a Rheodyne 7725i injection valve (5 mL and 20 mL loading loops) with a Waters 500 pump and a Waters 490E programmable wavelength detector operating at 214 nm and 280 nm. Linear peptides were purified using a Waters X-Bridge® C8 OBD^TM^ Column (5 µm, 30 × 150 mm) at a flow rate of 38 mL min^-1^. Folded peptides were purified using a Waters X-Bridge® C18 OBD^TM^ Semi-Prep Column (5 µm, 10 × 250 mm) at a flow rate of 5 mL min^-1^. Preparative HPLC used a mobile phase of 0.1% TFA in water (Solvent A) and 0.1% TFA in acetonitrile (Solvent B) and a linear gradient as specified**.**

### Ultra-Performance Liquid Chromatography-Mass Spectrometry

Ultra-Performance Liquid Chromatography-Mass Spectrometry (UPLC-MS) was performed on a Shimadzu 2020 UPLC-MS instrument with a Nexera X2 LC-30AD pump, Nexera X2 SPD-M30A UV/Vis diode array detector and a Shimadzu 2020 (ESI) mass spectrometer operating in positive ion mode. Separations were performed on a Waters Acquity BEH300 1.7 µm, 2.1 × 50 mm (C18) column at a flow rate of 0.6 mL min^-1^. All separations were performed using a mobile phase of 0.1 vol.% formic acid in water (solvent A) and 0.1 vol.% formic acid in MeCN (solvent B) using linear gradients over 5 min.

### Analytical High-Performance Liquid Chromatography

Analytical reversed-phase HPLC was performed on a Waters Alliance e2695 HPLC system equipped with a 2998 PDA detector (λ = 210–400 nm). Separations were performed on a Waters XBridge® Peptide BEH300 5 μm, 4.6 × 250 mm (C18) column at 40 ^o^C with a flow rate of 1.0 mL min^-1^. All separations were performed using a mobile phase of 0.1% TFA in water (Solvent A) and 0.1% TFA in MeCN (Solvent B) using linear gradients, unless otherwise specified. Analytical HPLC traces were processed where time 0 min refers to the start of the gradient.

### Mass Spectrometry

Low resolution mass spectra were recorded on a Shimadzu 2020 (ESI) mass spectrometer operating in positive and negative mode. High resolution mass spectra were recorded on a Bruker-Daltronics Apex Ultra 7.0 T Fourier transform (FTICR) mass spectrometer. All mass spectra provided in the manuscript and the Supporting Information are extracted from the entire UV peak in the UPLC-MS.

### Fmoc-Amino Acids and Carboxylic Acid Derivatives Used

Unless otherwise specified the following carboxylic acids (and derivatives) were used in the assembly of the linear peptides.


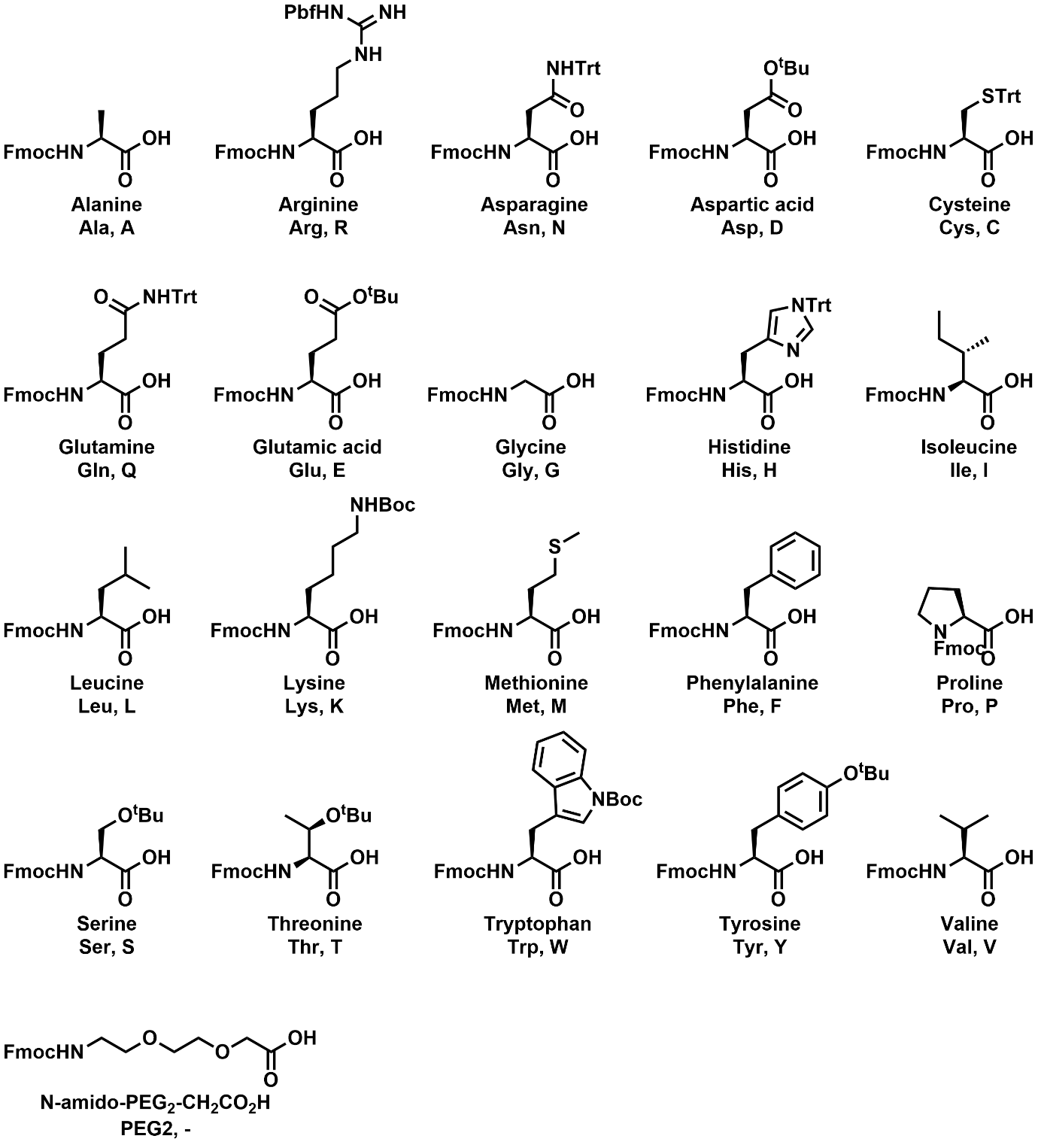


### Synthesis and Characterization of Linear aHSA Control

CTC resin (90 mg, 50 µmol, 0.56 mmol g^-1^) was loaded with Fmoc-Thr(OtBu)- OH according to general procedure **A**. The target peptide was then synthesized by iterative Fmoc solid-phase peptide synthesis (Fmoc SPPS) on the Syro I peptide synthesizer according to general procedure **C**, to generate the resin-bound sequence TCPDGYTRTNYYCRRDGCGSWCNGAERQQPCIRGPCCCDLTYRT. The resin was then treated with 20 vol.% piperidine in DMF (2 x 5 mL, 3 min) and washed with DMF (5 x 5 mL) and CH_2_Cl_2_ (5 x 5 mL). A portion (25 µmol) of the peptide was cleaved from resin by general procedure **D** and purified by preparative HPLC (1 to 50 vol.% MeCN in H_2_O with 0.1 vol.% TFA over 50 min), followed by lyophilization, afforded the pure peptide as a white fluffy solid (20.5 mg, 14%). **HRMS:** Calculated for [C_205_H_313_N_67_O_67_S_8_+H]^+^: 5045.1042, found 5045.1005. **LRMS:** (+ESI) *m/z* 1682.2 [M+3H]^3+^, 1261.9 [M+4H]^4+^, 1009.6 [M+5H]^5+^, 841.5 [M+6H]^6+^, 721.5 [M+7H]^7+^. **Analytical HPLC:** R_t_ = 17.6 min (0 to 50 vol.% MeCN in H_2_O with 0.1 vol.% TFA over 30 min, λ = 214 nm).

### Oxidative Folding and Characterization of Folded aHSA Control

### Supplementary Table 1: Buffer Screen for Oxidative Folding of aHSA Control

| Entry | Buffer | Additive | GSH | GSSG | Result |
| --- | --- | --- | --- | --- | --- |
| 1 | TRIS (20 mM) | NaCl (500 mM) | 1 mM | 0.02 mM | Folded (1 h). Impurities detected |
| 2 | NH_4_OAc (330 mM) | Gdn.HCl (500 mM) | 1 mM | 0.02 mM | Folded Well (16 h) |
| 3 | NaHCO_3_ (200 mM) | Urea (2M) | 1 mM | 0.02 mM | Folded Well (1 h) |
| 4 | (NH_4_)_2_SO_4_ (100 mM) | Arg (400 mM) | 1 mM | 0.02 mM | Folded (1 h). Impurities detected |
| 5 | TRIS (200 mM) | (NH_4_)_2_SO_4_ (100 mM), Arg (400 mM) | 1 mM | 0.02 mM | Folded (1 h). Impurities detected |
| 6 | NH_4_OAc (330 mM) | - | 1 mM | 0.02 mM | 3/4 disulfide bonds formed |
| 7 | NH_4_OAc (330 mM) | - | - | - | No folding |

### Buffer consisting of NaHCO_3_ (0.2 M), urea (2 M), and GSSG:GSH (0.2 mM:1 mM) was prepared and 4.0 mg of linear aHSA control was dissolved in rapid dilution buffer (1.6 mL; ultrapure milliQ, 50 mM TRIS, 150 mM NaCl, 6M Gdn.HCl) and added dropwise to the buffer (40 mL, final peptide concentration 0.1 mg/mL) in a 50 mL protein LoBind® falcon tube. The tube was sealed and left to stand at ambient temperature. The reaction mixture was monitored using analytical UPLCMS and MALDI until all linear material had converted to the folded mass (16 h). The crude folding mixture was purified using preparative reversed-phase HPLC (1 to 50 vol.% MeCN in H_2_O with 0.1 vol.% TFA over 50 min) to afford synthetic folded aHSA control as a white solid (2.1 mg, 52%).

### MALDI: Calculated for [C_205_H_305_N_67_O_67_S_8_+H]^+^: 5037.04159, found 5037.06600. LRMS: (+ESI) *m/z* 1679.6 [M+3H]^3+^, 1259.9 [M+4H]^4+^, 1008.0 [M+5H]^5+^, 840.2 [M+6H]^6+^, 720.2 [M+7H]^7+^. Analytical HPLC: R_t_ = 19.3 min (0 to 50 vol.% MeCN in H_2_O with 0.1 vol.% TFA over 30 min, λ = 214 nm).

###

### Synthesis and Characterization of Linear aMSA Control

CTC resin (90 mg, 50 µmol, 0.56 mmol g^-1^, Mimotopes) was loaded with Fmoc-Thr(OtBu)- OH according to general procedure **A**. The target peptide was then synthesized by iterative Fmoc solid-phase peptide synthesis (Fmoc SPPS) on the Syro I peptide synthesizer according to general procedure **C**, to generate the resin-bound sequence TCPDGQRDRGGCSGPYSCGGDNCCAYAA**AS**VYRGYSCKDT (**AS**: Fmoc-Ala-Ser(ψ^Me,Me^pro)-OH pseudoproline). The resin was then treated with 20 vol.% piperidine in DMF (2 x 5 mL, 3 min) and washed with DMF (5 x 5 mL) and CH_2_Cl_2_ (5 x 5 mL). A portion (25 µmol) of the peptide was cleaved from resin by general procedure **D** and purified by preparative HPLC (1 to 50 vol.% MeCN in H_2_O with 0.1 vol.% TFA over 50 min), followed by lyophilization, afforded the pure peptide as a white fluffy solid (8.1 mg, 7%).

**HRMS:** Calculated for [C_164_H_248_N_52_O_61_S_6_+H]^+^: 4116.63432, found 4116.62126. **LRMS:** (+ESI) *m/z* 1372.8 [M+3H]^3+^, 1029.8 [M+4H]^4+^, 824.1 [M+5H]^5+^. **Analytical HPLC:** R_t_ = 19.0 min (0 to 50 vol.% MeCN in H_2_O with 0.1 vol.% TFA over 30 min, λ = 214 nm).

### Oxidative Folding and Characterization of Folded aMSA Control

### Supplementary Table 2: Buffer Screen for Oxidative Folding of aMSA Control

| **Entry** | **Buffer** | **Additive** | **GSH** | **GSSG** | **Result** |
| --- | --- | --- | --- | --- | --- |
| 1 | TRIS (20 mM) | NaCl (500 mM) | 1 mM | 0.02 mM | Folded (4 h). Impurities detected |
| 2 | NH_4_OAc (330 mM) | Gdn.HCl (500 mM) | 1 mM | 0.02 mM | Folded Well (16 h) |
| 3 | NaHCO_3_ (200 mM) | Urea (2M) | 1 mM | 0.02 mM | Folded Well (16 h) |
| 4 | (NH_4_)_2_SO_4_ (100 mM) | Arg (400 mM) | 1 mM | 0.02 mM | Folded (1 h). Impurities detected |
| 5 | TRIS (200 mM) | (NH_4_)_2_SO_4_ (100 mM), Arg (400 mM) | 1 mM | 0.02 mM | Folded (1 h). Impurities detected |
| 6 | NH_4_OAc (330 mM) | - | 1 mM | 0.02 mM | 2/3 disulfide bonds formed |

### Buffer consisting of NaHCO_3_ (0.2 M), urea (2 M), and GSSG:GSH (0.2 mM:1 mM) was prepared and 4.3 mg of linear aMSA control was dissolved in rapid dilution buffer (1.7 mL; ultrapure milliQ, 50 mM TRIS, 150 mM NaCl, 6M Gdn.HCl) and added dropwise to the buffer (43 mL, final peptide concentration 0.1 mg/mL) in a 50 mL protein LoBind® falcon tube. The tube was sealed and left to stand at ambient temperature. The reaction mixture was monitored using analytical UPLCMS and MALDI until all linear material had converted to the folded mass (16 h). The crude folding mixture was purified using preparative reversed-phase HPLC (1 to 50 vol.% MeCN in H_2_O with 0.1 vol.% TFA over 50 min) to afford synthetic folded aMSA control as a white solid (3.0 mg, 70 %). MALDI: Calculated for [C_164_H_242_N_52_O_61_S_6_+H]^+^: 4110.587, found 4110.6066. LRMS: (+ESI) *m/z* 1370.7 [M+3H]^3+^, 1028.3 [M+4H]^4+^, 822.8 [M+5H]^5+^. Analytical HPLC: R_t_ = 18.1 min (0 to 50 vol.% MeCN in H_2_O with 0.1 vol.% TFA over 30 min, λ = 214 nm).

### Synthesis and Characterization of Linear aHSA-HAP Conjugate

CTC resin (90 mg, 50 µmol, 0.56 mmol g^-1^, Mimitopes) was loaded with Fmoc-Thr(OtBu)- OH according to general procedure **A**. The target peptide was then synthesized by iterative Fmoc solid-phase peptide synthesis (Fmoc SPPS) on the Syro I peptide synthesizer according to general procedure **C**, to generate the resin-bound sequence IHVTIPADLWDWINK-PEG2-TCPDGYTRTNYYCRRDGCGSWCNGAERQQPCIRGPCCCDLTYRT. The resin was then treated with 20 vol.% piperidine in DMF (2 x 5 mL, 3 min) and washed with DMF (5 x 5 mL), CH_2_Cl_2_ (5 x 5 mL) and DMF (5 x 5 mL) before acetylating the N-terminus with a solution of 10 vol.% Ac_2_O in pyridine (5 mL, 3 min). The resin was washed with DMF (5 x 5 mL) and CH_2_Cl_2_ (5 x 5 mL) and a portion (25 µmol) of the peptide was cleaved from resin by general procedure **D**. Purification by preparative HPLC (1 to 50 vol.% MeCN in H_2_O with 0.1 vol.% TFA over 50 min), followed by lyophilization, afforded the pure peptide as a white fluffy solid (7.2 mg, 4%). **HRMS:** Calculated for [C_300_H_453_N_89_O_92_S_8_+H]^+^: 7035.14310, found 7035.15933. **LRMS:** (+ESI) *m/z* 1759.4 [M+4H]^4+^, 1407.6 [M+5H]^5+^, 1173.3 [M+6H]^6+^, 1005.7 [M+7H]^7+^, 1005.7 [M+8H]^8+^, 780.5 [M+9H]^9+^. **Analytical HPLC:** R_t_ = 28.3 min (0 to 50 vol.% MeCN in H_2_O with 0.1 vol.% TFA over 30 min, λ = 214 nm).

### Oxidative Folding and Characterization of Folded aHSA-HAP Conjugate

### Supplementary Table 3: Buffer Screen for Oxidative Folding of aHSA-HAP Conjugate

| **Entry** | **Buffer** | **Additive** | **GSH** | **GSSG** | **Result** |
| --- | --- | --- | --- | --- | --- |
| 1 | TRIS (20 mM) | NaCl (500 mM) | 1 mM | 0.02 mM | No folding. Peptide precipitated |
| 2 | NH_4_OAc (330 mM) | Gdn.HCl (500 mM) | 1 mM | 0.02 mM | 3/4 disulfide bonds formed |
| 3 | NaHCO_3_ (200 mM) | Urea (2 M) | 1 mM | 0.02 mM | Folded (2 h). Precipitation observed |
| 4 | (NH_4_)_2_SO_4_ (100 mM) | Arg (400 mM) | 1 mM | 0.02 mM | Folded (2 h). Precipitation observed |
| 5 | NH_4_OAc (330 mM) | - | 1 mM | 0.02 mM | Folded (2 h). Precipitation observed |
| 6 | TRIS (200 mM) | (NH_4_)_2_SO_4_ (100 mM), Arg (400 mM) | 1 mM | 0.02 mM | Folded (2 h) |

### For entries 1-5, a precipitate was observed after 16 h of peptide standing in buffer which was determined measuring a reduction of soluble peptide concentration using NanoDrop UV-Vis spectroscopy. Buffer consisting of TRIS (0.2 M), (NH_4_)_2_SO_4_ (0.1 M), Arg (0.4 M), and GSSG:GSH (0.2 mM:1 mM) was prepared and 4.1 mg of linear aHSA-HAP conjugate was dissolved in rapid dilution buffer (1.64 mL; ultrapure milliQ, 50 mM TRIS, 150 mM NaCl, 6 M Gdn.HCl) and added dropwise to the buffer (41 mL, final peptide concentration 0.1 mg/mL) in a 50 mL protein LoBind® falcon tube. The tube was sealed and left to stand at ambient temperature. The reaction mixture was monitored using analytical UPLCMS and MALDI until all linear material had converted to the folded mass (16 h). The crude folding mixture was purified using preparative reversed-phase HPLC (1 to 50 vol.% MeCN in H_2_O with 0.1 vol.% TFA over 50 min) to afford synthetic folded aHSA-HAP conjugate as a white solid (1.2 mg, 29.5%). MALDI: Calculated for [C_300_H_445_N_89_O_92_S_8_+H]^+^: 7027.0805, found 7027.0902. LRMS: (+ESI) *m/z* 1757.3 [M+4H]^4+^, 1406.3 [M+5H]^5+^, 1171.9 [M+6H]^6+^, 1004.8 [M+7H]^7+^, 879.3 [M+8H]^8+^. Analytical HPLC: R_t_ = 22.1 min (0 to 50 vol.% MeCN in H_2_O with 0.1 vol.% TFA over 30 min, λ = 214 nm).

### Synthesis and Characterization of Linear aMSA-HAP Conjugate

CTC resin (90 mg, 50 µmol, 0.56 mmol g^-1^, Mimitopes) was loaded with Fmoc-Thr(OtBu)- OH according to general procedure **A**. The target peptide was then synthesized by iterative Fmoc solid-phase peptide synthesis (Fmoc SPPS) on the Syro I peptide synthesizer according to general procedure **C**, to generate the resin-bound sequence IHVTIPADLWDWINK-PEG2-TCPDGQRDRGGCSGPYSCGGDNCCAYAAASVYRGYSCKDT. The resin was then treated with 20 vol.% piperidine in DMF (2 x 5 mL, 3 min) and washed with DMF (5 x 5 mL), CH_2_Cl_2_ (5 x 5 mL) and DMF (5 x 5 mL) before acetylating the N-terminus with a solution of 10 vol.% Ac_2_O in pyridine (5 mL, 3 min). The resin was washed with DMF (5 x 5 mL) and CH_2_Cl_2_ (5 x 5 mL) and a portion (25 µmol) of the peptide was cleaved from resin by general procedure **D**. Purification by preparative HPLC (1 to 50 vol.% MeCN in H_2_O with 0.1 vol.% TFA over 50 min), followed by lyophilization, afforded the peptide as a white fluffy solid (32.10% Ala deletion, calculated through averaging integrations of the total ion chromatograms of [M+4H]4+, [M+5H]5+ and [M+6H]6+ charge states, 13.2 mg, 87.6%). **HRMS:** Calculated for [C_259_H_388_N_74_O_86_S_6_+H]^+^: 6106.67303, found 6106.72304. **LRMS:** (+ESI) *m/z* 1527.4 [M+4H]^4+^, 1222.0 [M+5H]^5+^, 1018.5 [M+6H]^6+^, 873.1 [M+7H]^7+^. **Analytical HPLC:** R_t_ = 26.2 min (0 to 50 vol.% MeCN in H_2_O with 0.1 vol.% TFA over 30 min, λ = 214 nm).

###

### Oxidative Folding and Characterization of Folded aMSA-HAP Conjugate

### Supplementary Table 4: Buffer Screen for Oxidative Folding of aMSA-HAP Conjugate

| **Entry** | **Buffer** | **Additive** | **GSH** | **GSSG** | **Result** |
| --- | --- | --- | --- | --- | --- |
| 1 | TRIS (20 mM) | NaCl (500 mM) | 1 mM | 0.02 mM | Folded (2 h). Precipitation observed |
| 2 | NH_4_OAc (330 mM) | Gdn.HCl (500 mM) | 1 mM | 0.02 mM | Folded (2 h). Precipitation observed |
| 3 | NaHCO_3_ (200 mM) | Urea (2M) | 1 mM | 0.02 mM | Folded (2 h). Precipitation observed |
| 4 | (NH_4_)_2_SO_4_ (100 mM) | Arg (400 mM) | 1 mM | 0.02 mM | Folded (2 h). Minor Precipitation observed |
| 5 | NH_4_OAc (330 mM) | - | 1 mM | 0.02 mM | Folded (2 h). Precipitation observed |
| 6 | TRIS (200 mM) | (NH_4_)_2_SO_4_ (100 mM), Arg (400 mM) | 1 mM | 0.02 mM | Folded (2 h) |

### For entries 1-5, a precipitate was observed after 16 h of peptide standing in buffer which was determined measuring a reduction of soluble peptide concentration using NanoDrop UV-Vis spectroscopy. Buffer consisting of TRIS (0.2 M), (NH_4_)_2_SO_4_ (0.1 M), Arg (0.4 M), and GSSG:GSH (0.2 mM:1 mM) was prepared and 4.4 mg of linear aMSA-HAP conjugate was dissolved in rapid dilution buffer (1.76 mL; ultrapure milliQ, 50 mM TRIS, 150 mM NaCl, 6M Gdn.HCl) and added dropwise to the buffer (44 mL, final peptide concentration 0.1 mg/mL) in a 50 mL protein LoBind® falcon tube. The tube was sealed and left to stand at ambient temperature. The reaction mixture was monitored using analytical UPLCMS and MALDI until all linear material had converted to the folded mass (16 h). The crude folding mixture was purified using preparative reversed-phase HPLC (1 to 50 vol.% MeCN in H2O with 0.1 vol.% TFA over 50 min) to afford synthetic folded aMSA-HAP conjugate as a white solid (1.4 mg, 31.8%).

### MALDI: Calculated for [C_259_H_382_N_74_O_86_S_6_+H]^+^: 6100.6261, found 6100.61601 LRMS: (+ESI) *m/z* 1525.9 [M+4H]^4+^, 1220.9 [M+5H]^5+^, 1017.6 [M+6H]^6+^. Analytical HPLC: R_t_ = 21.9 min (0 to 50 vol.% MeCN in H_2_O with 0.1 vol.% TFA over 30 min, λ = 214 nm).
